# Global Association between Thermophilicity and Vancomycin Susceptibility in Bacteria

**DOI:** 10.3389/fmicb.2016.00412

**Published:** 2016-03-31

**Authors:** Chayan Roy, Masrure Alam, Subhrangshu Mandal, Prabir K. Haldar, Sabyasachi Bhattacharya, Trinetra Mukherjee, Rimi Roy, Moidu J. Rameez, Anup K. Misra, Ranadhir Chakraborty, Ashish K. Nanda, Subhra K. Mukhopadhyay, Wriddhiman Ghosh

**Affiliations:** ^1^Department of Microbiology, Bose InstituteKolkata, India; ^2^Department of Microbiology, The University of BurdwanBurdwan, India; ^3^Division of Molecular Medicine, Bose InstituteKolkata, India; ^4^Department of Biotechnology, University of North BengalSiliguri, India; ^5^Department of Chemistry, University of North BengalSiliguri, India

**Keywords:** vancomycin susceptibility, thermophilic bacteria, MALDI-TOF mass spectrometry, NGS, peptidoglycan, D-alanine-d-alanine ligase

## Abstract

Exploration of the aquatic microbiota of several circum-neutral (6.0–8.5 pH) mid-temperature (55–85°C) springs revealed rich diversities of phylogenetic relatives of mesophilic bacteria, which surpassed the diversity of the truly-thermophilic taxa. To gain insight into the potentially-thermophilic adaptations of the phylogenetic relatives of Gram-negative mesophilic bacteria detected in culture-independent investigations we attempted pure-culture isolation by supplementing the enrichment media with 50 μg ml^−1^ vancomycin. Surprisingly, this Gram-positive-specific antibiotic eliminated the entire culturable-diversity of chemoorganotrophic and sulfur-chemolithotrophic bacteria present in the tested hot water inocula. Moreover, it also killed all the Gram-negative hot-spring isolates that were obtained in vancomycin-free media. Concurrent literature search for the description of Gram-negative thermophilic bacteria revealed that at least 16 of them were reportedly vancomycin-susceptible. While these data suggested that vancomycin-susceptibility could be a global trait of thermophilic bacteria (irrespective of their taxonomy, biogeography and Gram-character), MALDI Mass Spectroscopy of the peptidoglycans of a few Gram-negative thermophilic bacteria revealed that tandem alanines were present in the fourth and fifth positions of their muropeptide precursors (MPPs). Subsequent phylogenetic analyses revealed a close affinity between the D-alanine-D-alanine ligases (Ddl) of taxonomically-diverse Gram-negative thermophiles and the thermostable Ddl protein of *Thermotoga maritima*, which is well-known for its high specificity for alanine over other amino acids. The Ddl tree further illustrated a divergence between the homologs of Gram-negative thermophiles and mesophiles, which broadly coincided with vancomycin-susceptibility and vancomycin-resistance respectively. It was thus hypothesized that thermophilic Ddls have been evolutionarily selected to favor a D-ala-D-ala bonding. However, preference for D-ala-D-ala-terminated MPPs does not singlehandedly guarantee vancomycin susceptibility of thermophilic bacteria as the large and relatively-hydrophilic vancomycin molecule has to cross the outer membrane before it can inhibit peptidoglycan biosynthesis. Literature shows that many mesophilic Gram-negative bacteria also have D-ala-D-ala-terminated MPPs, but they still remain resistant to vancomycin due to the relative impermeability of their membranes. But the global vancomycin-susceptibility phenotype of thermophilic bacteria itself testifies that the drug crosses the membrane in all these cases. As a corollary, it seems quite likely that the outer membranes of thermophilic bacteria have some yet-unknown characteristic feature(s) that invariably ensures the entry of vancomycin.

## Introduction

Relatives of phylogenetically diverse mesophilic bacteria are known to be present in hot spring waters alongside the typically thermophilic and hyperthermophilic prokaryotes (Jimenez et al., 2012; Wemheuer et al., 2013; Chan et al., 2015; Menzel et al., 2015). Our ongoing exploration of the hot water microbiota of geographically- and physicochemically-discrete hydrothermal areas of India also discovered rich diversities of *Alpha*-, *Beta*- and *Gammaproteobacteria* occurring in conjunction with diverse members of *Bacteroidetes, Actinobacteria, Nitrospirae* and *Firmicutes*. Interestingly, in all the explored hot water communities, diversity of such phylogenetic relatives of mesophilic bacteria surpassed the diversity of the truly thermophilic *Aquificaea, Thermotogae* and *Thermodesulfobacteria* (see Results section below). So far as truly thermophilic bacteria (growth optima above 80°C Kristjansson and Stetter, 1992) are concerned, attributes like protein architecture (Kumar et al., 2000), DNA topology (Dekker et al., 2003), membrane lipid composition (Koga, 2012) etc. have been implicated as crucial to their extremely high temperature adaptation. In contrast, little insight is available on the molecular basis of bacterial adaptation below the hyperthermophilic boundary. And whatever little information is available is confined to Gram-positive *Firmicutes* like *Bacillus* (Volker et al., 1992; Schumann, 2003; Endo et al., 2006; Sikorski et al., 2008), *Geobacillus* (Shih and Pan, 2011; Tripathy and Maiti, 2014; Wang et al., 2014) and *Anoxybacillus* (Burgess et al., 2009; Paul et al., 2012; Goh et al., 2014). This is despite the fact that taxonomically-diverse bacteria are known to grow facultatively at temperatures between 50 and 80°C (often in addition to their mesophilic growths below 50°C) (Moreira et al., 2000; Alves et al., 2003; Rainey et al., 2003). In this scenario, our laboratory intended to isolate moderately-thermophilic bacteria outside the phylum *Firmicutes* so that they can be used to elucidate the biology of mid-temperature adaptation in Gram-negative bacteria. So we supplemented all the isolation media with the Gram-positive-specific antibiotic vancomycin (Watanakunakorn, 1984; Lundstrom and Sobel, 2000; Nailor and Sobel, 2009) in order to potentially eliminate *Firmicutes* and allow Gram-negative taxa to appear in the isolation plates. Interestingly, addition of vancomycin to the culture media caused complete destruction of all the tested hot water inocula at 55 as well as 30°C. However, parallel inoculation of vancomycin-free media yielded flocculent growth of mixed consortia at either temperature. This phenomenon held true for all the hot water inocula that were tested from various geothermal districts of India. Concurrently, all the Gram-negative bacteria that we could isolate in vancomycin-free media (plus their sibling strains that had been isolated earlier from other parts of the world) were found to be susceptible to vancomycin. Again, when we scrutinized the descriptions of Gram-negative thermophilic bacteria in the literature we found that at least 16 of them had been reported as vancomycin-susceptible, while a large majority had never been tested for this phenotype. All these observations collectively suggested that vancomycin-susceptibility could be a global trait of thermophilic bacteria, irrespective of their taxonomy, biogeography and Gram-character. Subsequently, MALDI Mass Spectroscopic (MS) analysis of the peptidoglycans of a few Gram-negative thermophilic bacteria revealed the occurrence of tandem alanines in the fourth and fifth positions of their muro-pentapetide precursors (MPPs), which again was the reported target of vancomycin-binding in Gram-positive *Firmicutes* (Barna and Williams, 1984; Reynolds, 1989). This proved that vancomycin killed Gram-negative bacteria by the same mode of action as that which kills Gram-positive bacteria. In conclusion, maximum likelihood (ML) phylogeny of the D-alanine-D-alanine ligase protein was reconstructed to elucidate the ecological and evolutionary basis of the wide spread of vancomycin-susceptibility in Gram-negative thermophilic bacteria.

## Materials and methods

### Sampling

For any given venting point, batches of 500 ml freshly-discharged hot water were passed through sterile 0.22 μm filters (4.7 cm radius). Filters were immediately put into cryovials containing 5 ml of either 50 mM:50 mM Tris:EDTA (TE, pH 7.8) or 15% Glycerol in 0.9% NaCl and transferred to the lab in dry ice. Upon reaching the lab the cryovials were stored at −20°C until further use, which was anyhow within 2 weeks from sampling. While the filters suspended in TE were used for the isolation of total environmental DNA, those put in glycerol stocks were used in various culture-based experiments (one filter per 80 ml of any medium).

### Isolation of environmental DNA

A given TE-suspended filter was cut into small pieces with sterile scissors and put back to the original cryovial. The vial was vortexed vigorously for 30 min, following which the filter shreds were discarded and the 5 ml TE distributed to five 1.5 ml microfuge tubes. All the five tubes were centrifuged at 10,800 g for 30 min, following which 900 μl TE was discarded from the top of each tube. The 100 μl TE remaining in each tube was vortexed vigorously for 15 min and the contents of all five tubes were pooled up into one microfuge tube. This pooled up 500 μl TE was again centrifuged at 10,800 g for 30 min, following which 400 μl was discarded from the top. The remaining 100 μl was presumably a suspension of all microbial cells recoverable from the 500 ml hot water sample in question. DNA was isolated from this 100 μl purported cell suspension by the QIAamp DNA Mini Kit (QIAGEN) following manufacturer's protocol.

### Media, culture conditions, and isolation of cultured-metagenomes

Chemoorganoheterotrophic growth experiments were performed in oligotrophic R2A medium while chemolithoautotrophic growth was checked in a modified basal mineral salt (MMS) medium [1 gm l^−1^ NH_4_Cl, 2 gm l^−1^ K_2_HPO_4_, 0.75 gm l^−1^ KH_2_PO_4_, 0.5 gm l^−1^ MgSO_4_ and 2 ml l^−1^ trace metals solution (Vishniac and Santer, 1957)] supplemented with 10 mM thiosulfate (MMST). Culturable diversity of chemoorganoheterotrophs or chemolithoautotrophs in a given hot water community was explored by incubating the glycerol stocked filters (at 55 as well as 30°C) in R2A or MMST broth respectively. From the resultant mixed consortia pure cultures were isolated by dilution plating 5 ml of cell suspensions in respective solid media. Vancomycin (50 μg ml^−1^) response of cultured consortia, or pure culture isolates, was tested in relevant media types at 55 as well as 30°C. For the isolation of metagenomes from the consortia cultured in R2A or MMST, 5 ml cell suspensions were harvested at appropriate time points and subjected to DNA preparation using the QIAamp DNA Mini Kit (QIAGEN) following manufacturer's protocol.

### Amplification of 16S rRNA gene fragments and sequencing by Ion PGM

V3 regions of all potential bacterial 16S rRNA genes present in an environmental or cultured metagenome were PCR-amplified by the “Fusion Primer Protocol” using *Bacteria*-specific universal oligonucleotides. In order to enable tandem sequencing of multiple PCR product pools on a single Ion chip a DNA template in question was subjected to PCR using a 16S forward primer prefixed with an Ion Torrent adapter and a unique sample-specific barcode or multiplex identifier in the following order in the 5′ to 3′ direction: (i) a 26-mer A-linker followed by a 4-mer A-linker key (bases represented in bold fonts), common for all sample primers, (ii) a 10-mer barcode unique to each sample primer followed by a common 3-mer barcode adaptor (all marked as stars), and finally (iii) the relevant domain-specific universal forward primer in its 5′ to 3′ direction (underlined bases). Reverse primers, in their turn, had (i) a common trP1 adapter (bases represented in italics), followed by (ii) the relevant domain-specific universal reverse primer in its 5′ to 3′ direction (underlined bases). Thus, V3 portions of all bacterial 16S rRNA genes present in a sample DNA were amplified using the forward primer 5′ – CCA TCT CAT CCC TGC GTG TCT CCG ACT CAG *** *** *** *** *CC TAC GGG AGG CAG CAG – 3′ and the reverse primer 5'-*CCT CTC TAT GGG CAG TCG GTG AT*A TTA CCG CGG CTG CTG G - 3′ (where underlined portions represent the universal primers 341f and 515r respectively).

Each 50 μl PCR reaction contained 10 μl template (corresponding to ~100 ng metagenomic DNA), 5 μl 10X KOD DNA polymerase buffer, 5 μl dNTP (0.25 mM each), 2 μl MgCl_2_ (25 mM), 1.5 μl (3%) DMSO, 3 μl each of the forward and reverse primer (0.3 μM each), 19.5 μl dH_2_O and 1 μl KOD hot start polymerase enzyme (Novagen, USA). PCR products were amplified for 30 cycles as follows: 94°C for 15 s, 65°C for 30 s and 68°C for 60 s. After amplification, all PCR products were electrophoresed on 2.5% w/v agarose gel, purified by size selection, and adjusted to final concentrations of 10 ng μl^−1^ using molecular grade water. PCR products from multiple samples were pooled up at equal concentrations for Ion PGM sequencing.

Before Ion PGM sequencing, size distribution and DNA concentration in the pooled-up amplicon mixture was examined using a Bioanalyzer 2100 (Agilent Technologies, USA). The mixed sample was adjusted to a final concentration of 26 pM and attached to the surface of Ion Sphere Particles (ISPs) using an Ion Onetouch 200 Template kit (Life Technologies, USA) according to the manufacturer's instructions. Manual enrichment of the resulting ISPs resulted in >95% templated-ISPs, which were then sequenced on Ion 316 Chips using the Ion PGM (Ion Express Template 200 chemistry) for 500 flows that gives an expected average read length of >220 bp. Sequencing was done upto such depths which ensured plateaus in rarefaction curves. Post sequencing, individual sequence reads were filtered by the PGM software to remove low quality and polyclonal sequences. Sequences matching the PGM 3′ adaptor were also automatically trimmed. All the data quality-filtered on the PGM were exported as fastq files for downstream applications.

The sequence files generated from PCR upon environmental DNA samples were deposited to the NCBI Sequence Read Archive (SRA) with the Run and BioProject accession numbers cited in Table 1. Similarly, sequence files obtained by PCR upon cultured metagenomes were deposited with the Run accession numbers cited in Table 2 and Supplementary Table S15 under the same BioProject as that used for sequences generated from environmental DNAs.

**Table 1:** **Bacterial taxonomic diversity detected in the hot waters of various geothermal vents surveyed in this study**.

**Geothermal district**	**Name of the spring**	**Geographical location of the hot springs (latitude/longitude)**	**Hydrothermal feature**	**Temp. and pH of the water**	**SRA run accession no. of the V3 sequence file**	**Number of OTUs identified**	**Phylum-level classification of OTUs**	**Link to the list of identified genera**
Sulfur- borax spring zone, Puga valley, Ladakh, J&K	Lotus Pond^*^ Center	Western part of the Puga valley, Ladakh, Jammu and Kashmir (33°13′46″ N/78°21′18″ E)	A hot water pool that is located on the bank of the Puga rivulet and has contiguous outflow with the latter	78–85°C pH 7-8	SRR1951799	331	Ubact, 32; *Prote*, 168; *Firm*, 38; *Actn*, 33; *Bact*, 24; *Cyan*, 12; D-T, 7; *Tmtg*, 4; *Def*, 2; *Acd*, 2; *Aq*, 2; *Chl*, 2; *Spir*, 1; *Cand Sach*, 1; *Ace*, 1; *Tmds*, 1; *Ignv*, 1.	Supplementary Table S2
	Lotus Pond-adjacent ebullition		A small hot water ebullition seated in between the Lotus Pond Center and the water flow of the Puga rivulet	70–80°C pH 7-7.5	SRR1951802	186	Ubact, 54; *Prote*, 72; *Actn*, 20; *Firm*, 17; *Bacts*, 10; *Acd*, 4; *Vera*, 3; *Armt*, 2; *Lats*, 1; *Cyan*, 1; *Cand Sach*, 1; *Chl*, 1.	Supplementary Table S3
	Shivlinga^*^		Fountain-type geyser	65–75°C pH 7-7.5	SRR1951804	80	Ubact, 24; *Prote*, 17; *Firm*, 14; D-T, 6; *Actn*, 5; *Bact*, 4; *Tmtg*, 2; *Aq*, 2; *Tmds*, 2; *Def*, 1; *Cyan*, 1; *Nitr*, 1; *Chl*, 1.	Supplementary Table S4
	PCPR_1	South-central part of Puga valley (33°13′27″ N/78°20′ 10 E″)	Hot water pool embedded in boratic deposits and valley-fill materials	70–75°C 7.5-8 pH	SRR1951805	135	Ubact, 7; *Prote*, 85; *Firm*, 28; *Actn*, 5; *Bact*, 5; *Cyan*, 1; *Tmtg*, 1; *Aq*, 1; *Tmds*, 1; *Ignv*, 1.	Supplementary Table S5
	PCPR_2	Northern part of Puga valley (33°13′25.51″ N/78°19′2.69″ E)	Fountain-type geyser	70–75°C pH 6.8-7.5	SRR1951815	188	Ubact, 17; *Prote*, 130; *Bact*, 23; *Actn*, 5; *Chl*, 5; *Cyan*, 2; *Firm*, 2; *Ignv*, 2; *Spir*, 1; *Acd*, 1.	Supplementary Table S6
Eastern Indian coalfields	Paniphala fountain	Paniphala, Burdwan, West Bengal (23°45′33.03″ N/86°58′54.28″ E)	Fountain-type geyser	55–65°C pH 6.8-8.0	SRR1951816	743	Ubact, 400; *Prote*, 184; *Actn*, 75; *Firm*, 45; *Bact*, 25; *Cyan*, 5; D-T, 3; *Fus*, 2; *Nitr*, 2; *Acd*, 1; *Ver*, 1	Supplementary Table S7
Eastern Indian lateritic belt	Agnikunda^†^	Bakreshwar, Birbhum, West Bengal (23°52′48.00″ N/87°22′12.00″ E)	Hot water pool partially confined by artificial embankments	70–85°C pH 6.0-7.0	SRR2016655	283	Ubact, 75; *Firm*, 67; D-T, 61; *Prote*, 23; *Tmtg*, 13; *Nitr*, 13; *Actn*, 12; *Atri*, 3; *Dict*, 3; *Chl*, 3; *Aq*, 3; *Bact*, 2; *Armt*, 2; *Ace*, 2; *Cyan*, 1	Supplementary Table S8
	Kharkunda^†^		Hot water pool partially confined by artificial embankments	55–65°C pH 7.5-8.5	SRR2016656	234	Ubact, 55; *Firm*, 63; D-T, 38; *Prote*, 33; *Tmtg*, 12; *Actn*, 11; *Nitr*, 9; *Atri*, 3; *Bact*, 2; *Cyan*, 2; *Armt*, 2; *Syn*, 1; *Ace*, 1; *Dict*, 1; *Chl*, 1	Supplementary Table S9

*Phylum-level classifications of the different OTU sets are shown; corresponding lists of identified genera are linked to respective supplementary tables. All V3 sequence files were deposited to the NCBI Sequence Read Archive (SRA) under the BioProject accession number PRJNA280244*.

* *The Shivlinga vent is just 50 m to the east of the Lotus Pond*.

† *The Agnikunda and Kharkunda are located within 10 m of each other*.

*Ubact, Unclassified Bacteria; Prote, Proteobacteria; Bact, Bacteroidetes; Firm, Firmicutes; Actn, Actinobacteria; Cyan, Cyanobacteria; D-T, Deinococcus-Thermus; Acd, Acidobacteria; Tmtg, Thermotogae; Def, Deferribacteres; Aq, Aquificae; Chl, Chloroflexi; Spir, Spirochaetes; Cand Sach, Candidatus Saccharibacteria; Ace, Acetothermia; Tmds, Thermodesulfobacteria; Ignv, Ignavibacteriae; Nitr, Nitrospirae; Fus, Fusobacteria; Armt, Armatimonadetes; Syn, Synergistetes; Dict, Dictyoglomi; Chrys, Chrysiogenetes; Atri, Atribacteria; Ver, Verrucomicrobia; Tenr, Tenericutes; Fibr, Fibrobacteres; Lats, Latescibacteria*.

**Table 2:** **Bacterial taxonomic diversity detected after incubating vent water inocula in R2A or MMST medium**.

**Vents**	**Growth response in R2A medium**				**Growth response in MMST medium**			
	**Incubation at 30**^**°**^**C**		**Incubation at 55**^**°**^**C**		**Incubation at 30**^**°**^**C**		**Incubation at 55**^**°**^**C**	
	**+V**	**-V**	**+V**	**-V**	**+V**	**-V**	**+V**	**-V**
Lotus Pond Center	0 OD_600_/480 h	0.8 OD_600_/16 h	0 OD_600_/480 h	0.8 OD_600_/12 h	7.0 pH; 0.05 OD_600_/168 h	6.0 pH; 0.3 OD_600_/72 h	7.0 pH; 0.05 OD_600_/168 h	5.0 pH; 0.4 OD_600_/36 h
		Total no. of OTUs detected: 30 (SRR1951817)		Total no. of OTUs detected: 58 (SRR1951818)		Total no. of OTUs detected: 68 (SRR1951819)		Total no. of OTUs detected: 136 (SRR1951820)
		Ubact, 3; *Firm*, 27		Ubact, 12; *Firm*, 19; *Prote*, 18; *Actn*, 6; *Bact*, 2; *Acd*, 1		Ubact, 18; *Firm*, 48; *Prote*, 1; *Actn*, 1		Ubact, 5; *Firm*, 104; *Prote*, 18; *Bact*, 4; *Actn*, 3; *Tmds*, 1; *Aq*, 1
		Supplementary Table S10A		Supplementary Table S10B		Supplementary Table S10C		Supplementary Table S10D
Lotus Pond-adjacent Vent	0 OD_600_/480 h	0.8 OD_600_/16 h	0 OD_600_/480 h	0.8 OD_600_/12 h	7.0 pH; 0.1 OD_600_/216 h	5.3 pH; 0.3 OD_600_/48 h	7.0 pH; 0.1 OD_600_/216 h	5.5 pH; 0.3 OD_600_/36 h
		Total no. of OTUs detected: 85 (SRR1951983)		Total no. of OTUs detected: 128 (SRR1951984)		Total no. of OTUs detected: 134 (SRR1951988)		Total no. of OTUs detected: 62 (SRR1951992)
		Ubact, 3; *Firm*, 81; *Prote*, 1		Ubact, 2; *Firm*, 122; *Prote*, 3; *Actn*, 1		Ubact, 11; *Firm*, 120; *Prote*, 3		Ubact, 1; *Firm*, 54; *Prote*, 4; *Actn*, 3
		Supplementary Table S11A		Supplementary Table S11B		Supplementary Table S11C		Supplementary Table S11D
Shivlinga Vent	0 OD_600_/480 h	0.8 OD_600_/16 h	0 OD_600_/480 h	0.8 OD_600_/12 h	7.0 pH; 0.1 OD_600_/120 h	6.0 pH; 0.3 OD_600_/36 h	7.0 pH; 0.1 OD_600_/120 h	5.5 pH; 0.4 OD_600_/16 h
		Total no. of OTUs detected: 54 (SRR1952883)		Total no. of OTUs detected: 51 (SRR1952893)		Total no. of OTUs detected: 320 (SRR1952904)		Total no. of OTUs detected: 51 (SRR1952937)
		Ubact, 11; *Actn*, 24; *Prote*, 12; *Firm*, 3; *Cyan*, 1; *Aq*, 1; D-T, 1; *Tmds*, 1		Ubact, 21; D-T, 21; *Prote, 4*; *Actn*, 1; *Aq*, 1; *Firm, 1*; *Tmds*, 1; *Chl*, 1		Ubact, 21; *Prote*, 184; *Actn*, 42; *Firm*, 39; *Bact*, 16; D-T, 5; *Cyan*, 4; *Tmds*, 2; *Chl*, 2; *Fus*, 1; *Armt*, 1; *Cand Sach*, 1; *Tmtg*, 1; *Aq*, 1		Ubact, 3; *Firm*, 45; *Prote*, 2; *Actn*, 1
		Supplementary Table S12A		Supplementary Table S12B		Supplementary Table S12C		Supplementary Table S12D
Paniphala Fountain	0 OD_600_/480 h	0.8 OD_600_/12 h	0 OD_600_/480 h	0.8 OD_600_/12 h	7.0 pH; 0.1 OD_600_/216 h	5.5 pH; 0.4 OD_600_/72 h	7.0 pH; 0.1 OD_600_/216 h	5.5 pH; 0.4 OD_600_/48 h
		Total no. of OTUs detected: 61 (SRR1952938)		Total no. of OTUs detected: 55 (SRR1954984)		Total no. of OTUs detected: 108 (SRR1954986)		Total no. of OTUs detected: 46 (SRR1954987)
		Ubact, 2; *Firm*, 50; *Prote*„ 6; *Actn*, 2; D-T, 1		Ubact, 2; *Firm*, 33; *Prote*, 17; *Actn*, 3		Ubact, 8; *Firm*, 97; *Actn*, 1; *Bact*, 1; *Cand Sach*, 1		Ubact, 1; *Firm*, 44; *Prote*, 1
		Supplementary Table S13A		Supplementary Table S13B		Supplementary Table S13C		Supplementary Table S13D
Agnikunda	0 OD_600_/480 h	0.8 OD_600_/16 h	0 OD_600_/480 h	0.8 OD_600_/16 h	7.0 pH; 0.05 OD_600_/216 h	5.0 pH; 0.4 OD_600_/48 h	7.0 pH; 0.1 OD_600_/216 h	6.5 pH; 0.3 OD_600_/96 h
		Total no. of OTUs detected: 53 (SRR2016659)		Total no. of OTUs detected: 79 (SRR2016660)		Total no. of OTUs detected: 78 (SRR2016657)		Total no. of OTUs detected: 112 (SRR2016658)
		Ubact, 3; *Firm*, 50		Ubact, 1; *Firm*, 78		Ubact, 2; *Firm*, 70; D-T, 2; *Cyan*, 1; *Prote*, 1; *Aq*, 1; *Tmds*, 1		Ubact, 12; *Firm*, 49; *Prote, 29; Actn*, 8; D-T, 4; *Tmtg*, 3; *Nitr*, 2; *Bact*, 1; *Cyan*, 1; *Armt*, 1; *Atri*, 1; *Dict*, 1
		Supplementary Table S14A		Supplementary Table S14B		Supplementary Table S14C		Supplementary Table S14D

*End points of growths from where cultured metagenomes were prepared for PCR amplification and sequencing are described by the OD_600_ and pH of the spent media followed by the incubation time. V3 sequence files were deposited to the NCBI SRA (see accession numbers in parenthesis) under the BioProject PRJNA280244. Phylum-level classifications of the different OTU sets are shown here; genus-level classifications are linked out to corresponding supplementary tables*.

*+V, growth medium supplemented with vancomycin (50 μg ml^−1^); -V, no vancomycin in growth medium*.

*Ubact, Unclassified Bacteria; Prote, Proteobacteria; Bact, Bacteroidetes; Firm, Firmicutes; Actn, Actinobacteria; Cyan, Cyanobacteria; D-T, Deinococcus-Thermus; Acd, Acidobacteria; Tmtg, Thermotogae; Aq, Aquificae; Chl, Chloroflexi; Cand Sach, Candidatus Saccharibacteria; Tmds, Thermodesulfobacteria; Fus, Fusobacteria; Armt, Armatimonadetes; Nitr, Nitrospirae;; Dict, Dictyoglomi; Atri, Atribacteria*.

### Taxonomic diversity estimation

Raw V3 region-specific reads were first filtered for high quality value (QV 20) and length threshold of 100 bp. Selected reads were then converted to fasta from fastq using Fastx_toolkit (v0.0.13.2). Operational taxonomic units (OTUs) were created at 97% identity level using the various modules of the UPARSE (Edgar, 2013) OTU clustering methods. Singletons were discarded from the data sets. A Perl script was used to get the ACE, Chao, Shannon and Simpson's diversity and abundance indices. Rarefaction analysis was done using a module of R-package and graph was created for reads taking part in OTU formation vs. number of OTUs formed. All consensus sequences generated for a given dataset were taxonomically classified with the help of the “RDP Classifier” located at http://rdp.cme.msu.edu/classifier/classifier.jsp.

### Peptidoglycan isolation

Peptidoglycans of various bacterial strains were prepared by methods described earlier (Komagata and Suzuki, 1988; Schumann, 2011). For vancomycin-untreated cells, peptidoglycan was prepared directly from mid-log phase cultures (OD_600_ = 0.6), whereas for vancomycin-treated counterparts the antibiotic (300 μg ml^−1^) was added to actively growing cultures when their OD_600_ was 0.3–0.4. The latter sets were then incubated for four more hours before cells were harvested.

Cells were collected by centrifugation and the cell pellet (2 gm wet weight) was washed twice with 5 ml phosphate buffer (0.05 M, pH 7.2). Then the cell pellet was resuspended in 6 ml 0.05 M phosphate buffer (pH 7.2) and cells were disrupted by sonication on ice using 20 s pulse for 4 times. The cell lysate was centrifuged at 1800 g for 10 min and the supernatant was transferred to a fresh centrifuge tube. It was then centrifuged at 12,000 g for 1 h. Supernatant was discarded and pellet was resuspended in 5 ml phosphate buffer. 1 ml of 5% sodium dodecyl sulfate was added and incubated at 100°C for 40 min, followed by centrifugation at 12,000 g for 30 min at 30°C. Then the pellet was washed four to five times with 5 ml 60°C distilled water. It was then washed with 5 ml 0.05 M phosphate buffer (pH 7.6). The pellet was then resuspended in 2 ml phosphate buffer (0.05 M, pH 7.6) and 100 μl pronase E (1 mg ml^−1^) was added. The soup was then incubated at 37°C for 2 h. Pellet was collected by centrifugation at 12,000 g for 30 min. It was further washed twice 2 ml phosphate buffer (0.05 M, pH 7.6). After that, pellet was resuspended in 2 ml of 5% TCA (Trichloroacetic Acid) and boiled at 100°C for 20 min. The suspension was cooled at room temperature and transferred to glass centrifuge tubes. It was then centrifuged at 12,000 g for 30 min. Pellet was further washed thrice with 2 ml phosphate buffer (0.05 M, pH 7.6), once each with 2 ml ethanol (95%) and 2 ml diethyl ether (99%), and finally air dried at 60°C for 3 h before further analysis.

### MALDI-MS

Extracted peptidoglycans were digested with lysozyme (40 mg ml^−1^) for 2 h at 37°C, following which deactivation was done for 20 min at 70°C. Digested products were lyophilized, resuspended in 100 μl 99% methanol, and directly used for MALDI-MS without any more purification. DHA was used as the MALDI matrix. MALDI-MS was carried out using an AutoFlex II tandem time of flight (TOF/TOF) MALDI-mass spectrometer (Bruker Daltonics) equipped with a pulsed N_2_ laser (λ-337 nm, 50 Hz). The machine was calibrated for reflector mode mass spectra using a mixture of standard peptides (having mass 750 to 3150) in the positive ion mode. MS spectra were analyzed using the Flex Analysis software V2.4.

### Phylogeny

ML trees were constructed using MEGA6 (Tamura et al., 2013) plus PhyML (Guindon et al., 2010). The best substitution model used for likelihood analysis (general time reversible and gamma) was selected by Bayesian as well as corrected Akaike information criteria. After the starting NJ tree was obtained heuristic searches for likelihood were performed using the Nearest–Neighbor–Interchange as well as Close–Neighbor–Interchange branch swapping algorithms.

## Results and discussion

### Rich diversity of phylogenetic relatives of mesophilic bacteria in circumneutral hot springs

Over the past few years we have investigated the taxonomic diversity (species richness) of the aquatic bacterial community of several circumneutral hot springs of Northern and Eastern India by analyzing amplified 16S rRNA gene fragments. V3 regions of all potential bacterial 16S rRNA genes present in the total environmental DNA isolated from thermal water samples were PCR-amplified using *Bacteria*–specific primers and sequenced by Ion PGM. Since many of the explored springs had similar taxonomic compositions only eight representative communities (five from Northern and three from Eastern India) are described in this paper. Statistics of clustering operational taxonomic units (OTUs) from the Ion PGM readsets is given in Supplementary Table S1. Table 1 shows the spring identities and the phylum-level classifications of their respective OTU sets. List of genera identified in these eight OTU sets are separately given in Supplementary Table S2 through Supplementary Table S9.

Notwithstanding the discrete locations and physicochemical characters of the studied springs, taxonomic structure of their hot water communities was unified by the occurrence of several such bacterial taxa that are otherwise unexpected in high-temperature habitats. While most of the communities encompassed maximum OTUs from the *Alpha, Beta*, and *Gamma* subclasses of *Proteobacteria*, the typical thermophilic phyla *Aquificae, Thermotogae* and *Thermodesulfobacteria* had very few OTUs affiliated to them (Table 1). The only exceptions to this trend were the Agnikunda and Kharkunda vents where taxonomic diversity was dominated by *Firmicutes*. Concurrent to these observations, a close inspection of the lists of genera identified in the described hot water communities revealed several such bacteria that have no report of laboratory growth above 45°C. While the hot water community of Agnikunda encompassed the lowest proportion (~52%) of such apparently-mesophilic genera, that of the Paniphala vent had the highest percentage (89%). So far as the actual numbers were concerned, the Lotus Pond Center (84) had the maximum count of supposedly-mesophilic genera, followed by Paniphala (81). In contrast, PCPR 1 and Shivlinga had the lowest number (11 and 15 respectively) of such genera, presumably because these vents as such had the lowest overall count of OTUs and genera.

In this scenario we sought to know how this large variety of purportedly-mesophilic genera survived in these high temperature habitats. First it was imperative to check whether they could at all grow at high temperatures or were only thermo-enduring entities. Alternatively, it was also plausible that many of them were stochastically introduced into these habitats in recent times and were not at all equipped to cope with thermal stress. Since only pure culture isolates could answer these queries we attempted to get the same in chemoorganoheterotrophic (R2A) as well as chemolithoautotrophic [modified minimal salts supplemented with thiosulfate, MMST (Ghosh and Roy, 2006)] media at various incubation temperatures between 30 and 70°C.

### Preponderance of *Firmicutes* in R2A plates

Strains related to *Bacillus, Geobacillus, Anoxybacillus* and *Brevibacillus* crowded all the R2A isolation plates incubated at temperatures ≥50°C. Consequently, very few non-*Firmicutes* (e.g., strains of known moderate-thermophiles like *Thermomonas, Porphyrobacter, Meiothermus* etc.) could be obtained even after several rounds of isolation from the various inoculum samples. May be, during high-temperature growth in R2A the *Firmicutes* out-competed other moderate-thermophiles by virtue of their metabolic versatility, faster growth rate and better thermal adaptations. So far as isolation in MMST was concerned, all the tested inocula acidified the enrichment broths and produced sulfate with concomitant disappearance of thiosulfate. However, only a few moderately-thermophilic *Proteobacteria* (e.g., *Thermithiobacillus tepidarius*) kept recurring when the enriched broths were plated in MMST-agar to obtain single colonies. In addition, a host of such apparently thermo-enduring *Paracoccus* strains got isolated (in MMST at 37°C) that despite failing to grow above 45°C did not lose substantial viability (measured by drop in CFU count of experimental cultures) even at 60°C over an exposure period of ~4–6 h. Now, our prime objective was to isolate thermophilic siblings of the reportedly-mesophilic Gram-negative bacteria listed in Supplementary Table S2 through Supplementary Table S9. Although success of such undertakings depended on the actual cultivability of the unexpected bacteria in question, failures could never ascertain whether the negative results were due to short-comings or intrinsic-limitations of the isolation procedures or whether the Gram-negative mesophiles surfacing in diversity analyses were not thermally-adapted at all. So we initiated another round of isolation by supplementing all media types with vancomycin so that *Firmicutes* were eliminated and Gram-negative taxa got a better chance to appear in the isolation plates.

### Eradication of culturable diversity by vancomycin

Supplementing R2A as well as MMST with 50 μg ml^−1^ vancomycin caused complete destruction of the corresponding culturable-diversities of all the explored hot water communities. However, to keep it brief, the data from three North Indian and two East Indian vents will be presented in detail. As shown in Table 2, zero or near-zero OD_600_ values were registered for all spent vancomycin-containing media after prolonged incubation at 55 as well as 30°C. No production of sulfuric acid in spent vancomycin-containing MMST media was taken as the main evidence of destruction of the culturable chemolithotrophic diversity. Concurrently-recorded CFU counts were always < 10^2^ ml^−1^ of the spent media. This appeared to tally with spontaneous mutation rates (John et al., 1998) as parallel vancomycin-free cultures yielded rich growth of mixed consortia at 55 as well as 30°C. For all the tested inocula, OD_600_ of vancomycin-free R2A reached 0.8 (CFU counts ~10^8^ ml^−1^ of spent medium) within 12–16 h of incubation, while that of vancomycin-free MMST reached 0.3–0.4 (CFU counts 10^4^–10^5^ ml^−1^ of spent medium) with concomitant acidification of spent media within 16–72 h of incubation. With regard to growth in MMST it must be appreciated that the observed OD_600_ values were not exclusively due to sulfur-oxidizing chemolithotrophs. Even though their active presence was evidenced by sulfuric acid production, the final cell masses recovered from spent MMST media were quite likely to contain organoheterotrophic secondary consumers.

The above data clearly implied that all the bacteria growing in the two vancomycin-free media types, irrespective of their taxonomic identity and Gram-property, were susceptible to this so-called Gram-positive-specific antibiotic. To know the taxonomic identity of these cultured consortia we isolated their total genomes, amplified the V3 regions of all 16S rRNA genes present therein, and sequenced the amplicon pools by Ion PGM. The obtained V3 readsets were analyzed by OTU-clustering, statistics of which are given in Supplementary Table S1. Table 2 shows the phylum-level classification of the respective OTU sets.

Corroborating the outcome of the isolation experiments, almost all the vancomycin-free cultured consortia (irrespective of the media type) encompassed maximum OTUs from *Firmicutes* (albeit after the unclassifiable ones). Nevertheless, *Actinobacteria, Deinococcus*-*Thermus* and *Proteobacteria* had maximum OTUs in the consortia obtained by incubating the Shivlinga Vent water in R2A at 30 and 55°C, and MMST at 30°C respectively. The most interesting attribute of these datasets was the affiliation of a large number of OTUs to Gram-negative phyla such as *Proteobacteria, Bacteroidetes, Deinococcus*-*Thermus, Thermodesulfobacteria, Acidobacteria, Cyanobacteria*, and also *Actinobacteria*, which has Gram-negative as well as positive members. The lists of genera identified in these OTU sets (Supplementary Table S10 through Supplementary Table S14) also showed that the vancomycin-free cultured consortia encompassed at least one (for Lotus Pond Center inoculum incubated in MMST at 30°C) to at most 46 (for Shivlinga inoculum incubated in MMST at 30°C) Gram-negative genera. Only when the Lotus Pond Center inoculum was incubated in R2A at 30°C, or the Agnikunda inoculum was incubated in R2A at 30 or 55°C, the resultant consortium encompassed no Gram-negative genus.

The above data summarily indicated that the Gram-negative components of hot water communities were as susceptible to vancomycin as their Gram-positive counterparts. The wide taxonomic as well as geographic spread of these community analyses further hinted that the association between vancomycin-susceptibility and thermal adaptation could well be a global phenomenon. Significantly again, this comprehensive vancomycin-susceptibility of all taxonomic- and Gram-types was not detected when the above experiments were repeated with mesophilic (*in situ* temperature 30°C) lake water inocula (see Supplementary Tables S15, S15A through Supplementary Table S15D). This buttressed our assumption that the phenomenon was indeed an exclusive hallmark of hydrothermal communities. Subsequently, to cross-examine this supposition, vancomycin challenge was extended to all the hot spring isolates that were there at our disposal. On top of that we also scrutinized the species descriptions of several Gram-negative thermophilic bacteria in the literature to see whether any information existed about their vancomycin response.

### Vancomycin-susceptibility is widespread among gram-negative thermophiles

All the current hot-spring isolates died (showing near-zero CFU counts) in the presence of 50 μg ml^−1^ vancomycin at both higher and lower incubation temperatures. Susceptible phenotype was also exhibited by the tested siblings strains of the present isolates reported from other parts of the world. Although the strains in question took negative Gram stain, their response to vancomycin challenge was exactly same as that of the *Bacillus* controls. On the other hand, mesophilic Gram-negative *Alpha*-, *Beta*- and *Gammaproteobacteria* like *Pseudaminobacter salicylatoxidans* KCT001, *Advenella kashmirensis* WT001 and *E*. *coli* BL21 did not show any growth perturbation in the presence of vancomycin. Relevant growth experiment data for some of these organisms are shown in Table 3.

**Table 3:** **Pure culture isolates of Gram negative bacteria tested in the present study for their vancomycin response**.

**Name of the hydrothermal isolate**	R2A^*^				MMST^**^			
	**28**^**°**^**C**		**55**^**°**^**C**		**28**^**°**^**C**		**55**^**°**^**C**	
	**+V**	**−V**	**+V**	**−V**	**+V**	**−V**	**+V**	**−V**
*Thermomonas hydrothermalis* SLCR1D	OD_600_ = 0.00/10 d	OD_600_ = 0.52/24 h	OD_600_ = 0.00/10 d	OD_600_ = 0.72/24 h	NA	NG	NA	NG
*Thermomonas hydrothermalis* DSM 14834	OD_600_ = 0.00/10 d	OD_600_ = 0.62/24 h	OD_600_ = 0.00/10 d	OD_600_ = 0.88/24 h	NA	NG	NA	NG
*Porphyrobacter cryptus* SLCR2	OD_600_ = 0.00/10 d	OD_600_ = 0.80/24 h	OD_600_ = 0.00/10 d	OD_600_ = 0.86/24 h	NA	NG	NA	NG
*Porphyrobacter cryptus* DSM 12079	OD_600_ = 0.00/10 d	OD_600_ = 0.84/24 h	OD_600_ = 0.00/10 d	OD_600_ = 0.88/24 h	NA	NG	NA	NG
*Meiothermus* sp. RP^†^	NA	NG	OD_600_ = 0.00/10 d	OD_600_ = 0.58/24 h	NA	NG	NA	NG
*Meiothermus* sp. TP^‡^	NA	NG	OD_600_ = 0.00/10 d	OD_600_ = 0.64/24 h	NA	NG	NA	NG
*Thermithiobacillus tepidarius* SMMA_11	NA	NG	NA	NG	OD_600_ = 0.00, pH 7.0/7 d	OD_600_ = 0.26, pH 5.7/24 h	OD_600_ = 0.00, pH 7.0/7 d	OD_600_ = 0.29, pH 5.7/24 h
*Paracoccus* sp.^$^ SMMA_7	OD_600_ = 0.00/10 d	OD_600_ = 0.92/24 h	NA	NG	OD_600_ = 0.00, pH 7.0/7 d	OD_600_ = 0.25, pH 6.0/24 h	NA	NA
*Paracoccus* sp.^$^ SMMA_5	OD_600_ = 0.00/10 d	OD_600_ = 0.92/24 h	NA	NG	OD_600_ = 0.00, pH 7.0/7 d	OD_600_ = 0.25, pH 6.0/24 h	NA	NA
*Bacillus licheniformis* SWCR_1/2X50_9	OD_600_ = 0.00/10 d	OD_600_ = 0.63/24 h	OD_600_ = 0.00/10 d	OD_600_ = 0.82/24 h	NA	NG	NA	NG
*Bacillus sp.* SWCR_604	OD_600_ = 0.00/10 d	OD_600_ = 0.69/24 h	OD_600_ = 0.00/10 d	OD_600_ = 0.77/24 h	NA	NG	NA	NG
*Pseudaminobacter salicylatoxidans* KCT001	OD_600_ = 0.53/48 h	OD_600_ = 0.52/48 h	NA	NG	OD_600_ = 0.27, pH 6.0/48 h	OD_600_ = 0.25, pH 6.0/48 h	NA	NG
*Advenella kashmirensis* WT001	OD_600_ 0.48/24 h	OD_600_ = 0.46/24 h	NA	NG	OD_600_ = 0.30, pH 6.0/24 h	OD_600_ 0.32, pH 6.0/24 h	NA	NG
*E. coli* BL21	OD_600_ = 0.57/24 h	OD_600_ = 0.55/24 h	NA	NG	NA	NG	NA	NG
*Paracoccus patotrophus* LMG 4218	OD_600_ = 0.85/24 h	OD_600_ = 0.88/24 h	NA	NG	OD_600_ = 0.29, pH 6.0/24 h	OD_600_ = 0.29, pH 6.0/24 h	NA	NG
*Paracoccus thiocyanatus* MTCC 7821	OD_600_ = 0.78/24 h	OD_600_ = 0.79/24 h	NA	NG	OD_600_ = 0.27, pH 6.0/24 h	OD_600_ = 0.26, pH 6.0/24 h	NA	NG

*+V, growth medium supplemented with vancomycin (50 μg ml-1), -V, no vancomycin in growth medium*.

*NG, No intrinsic growth in the relevant medium or temperature; NA, Not applicable*.

† *94% 16S rRNA gene sequence similarity with M. cateniformans, M. taiwanensis M. rubber and other Meiothermus spp*.

‡ *98% 16S rRNA gene sequence similarity with M. cateniformans, M. taiwanensis M. rubber and other Meiothermus spp*.

$ *These two Paracoccus strains had 100% 16S rRNA gene sequence similarity among themselves and 97% similarity with a host of Paracoccus spp. Importantly however, SMMA_7 was isolated from 68°**C** water while SMMA_5 was isolated from 80°C water*.

* *Bacteria were first grown in R2A broths without any antibiotic selection (seed cultures); subsequently 2% of these mid-log phase (OD_600_ = 0.6) seed cultures were transferred to R2A broths, which contained vancomycin (50 μg ml^−1^) or not, as warranted by the test in hand*.

** *Bacteria were first grown in MST broths (initial pH 7.0) without any antibiotic selection (seed cultures); subsequently 2% of these mid-log phase (OD_600_ = 0.15, pH 6.5) seed cultures were transferred to MST broths, which contained vancomycin (50 μg ml^−1^) or not, as warranted by the test in hand*.

The most remarkable of all these observations was the susceptibility of the thermo-enduring *Paracoccus* isolates. They were all killed by 50 μg ml^−1^ vancomycin at the same time as standard strains of *Paracoccus pantotrophus* (LMG 4218) and *Paracoccus thiocyanatus* (MTCC 7821) isolated from various mesophilic habitats remained unaffected (Table 3). This evoked the conjecture that vancomycin-susceptibility of Gram-negative bacteria could be a function of high temperature exposure rather than actual growth efficiency at elevated temperatures. In tandem with these experiments we also scrutinized the descriptions of Gram-negative thermophilic bacteria in the literature and found at least 16 such species that were reported as susceptible to vancomycin (Table 4). For obvious reasons, Gram-negative bacteria are seldom tested for vancomycin response; hence scarcity of such data was understandable. In such a scenario, the taxonomy and biogeography of the bacteria listed in Table 4 could be considered diverse enough to implicate vancomycin susceptibility as a global trait of Gram-negative thermophiles.

**Table 4:** **Gram negative thermophilic bacteria found to be susceptible to vancomycin**.

**Organism**		**Geography and Ecology of the isolate**	**Growth temperature range**	**Temperature(s) and concentration(s) of vancomycin test**	**References**
**Vancomycin response tested during chemoorganotrophic growth**					
*Prote*^†^	*Thermomonas hydrothermalis* SLCR1D	Hot water of the Shivlinga Fountain, Northern Puga valley, Ladakh, India	30–60°C	55°C and 30°C (50 μg ml^−1^)	This study
*Thermomonas hydrothermalis* DSM 14834	Water of a hot spring at São Gemil in Central Portugal	30–60°C	55°C and 30°C (50 μg ml^−1^)	This study, (Alves et al., 2003)
*Porphyrobacter cryptus* SLCR2	Hot water of the Shivlinga fountain, Northern Puga valley, Ladakh, India	30–55°C	55°C and 30°C (50 μg ml^−1^)	This study
*Porphyrobacter cryptus* DSM 12079	Run-off of a hot spring located at Alcafache in Central Portugal	30–55°C	55°C and 30°C (50 μg ml^−1^)	This study, Rainey et al., 2003
*Rubellimicrobium thermophilum* DSM 16684	Colored biofilms growing on paper machines and pulp dryers	28–56°C	56°C (Disc diffusion method)	Denner et al., 2006
*Deinococcus-Thermus*	*Meothermus* spp.	Hot water of the Paniphala Fountain, Eastern Coalfields, Paniphala, West Bengal	50–60°C	60°C (50 μg ml^−1^)	This study
*Thermus filiformis* ATCC 43280	Pool water from a hot spring in the Waimangu thermal valley, New Zealand	37–80°C	70°C (20 μg ml^−1^)	Hudson et al., 1984, 1987
*Thermus aquaticus* ATCC 25104	Algal-bacterial mat from Mushroom Spring, Lower Geyser Basin, Yellowstone National Park, USA	40–79°C	70°C (20 μg ml^−1^)	Brock and Freeze, 1969; Hudson et al., 1987
*Thermus thermophilus* ATCC 27634	Thermal water of a hot spring located at Mine, Shizuoka Prefecture, Japan.	47–82°C	70°C (20 μg ml^−1^)	Oshima and Imahori, 1974; Hudson et al., 1987; Williams et al., 1995
*Deferribacteres*	*Deferribacter thermophiles* ACM 5093	A well in the Beatrice oil field located in the British sector of the North Sea near the coast of Scotland	50–65°C	60°C (150 μg ml^−1^)	Greene et al., 1997
*Flexistipes sinusarabici*	Brine water samples of the Atlantis II deep of the Red Sea at a depth of 2000m	30–53°C	50°C (150 μg ml^−1^)	Fiala et al., 1990
*Calditerrivibrio nitroreducens*	Hot spring water from Yumata, Nagano, Japan	30–65°C	55 30°C (100 μg ml^−1^)	Iino et al., 2008
*Dic*^#^	*Dictyoglomus thermophilum* ATCC 35947	Tsuetate Hot Spring, Kumamoto Prefecture, Japan	50–80°C	73°C (100 μg ml^−1^)	Saiki et al., 1985
*Act*^$^	*Acidothermus cellulolyticus* ^*^ ATCC 43068	Upper Norris Geyser basin, Yellowstone National Park, USA	37–70°C	55°C (1 μg ml^−1^)	Mohagheghi et al., 1986
*Thermotogae*	*Fervidobacterium gondwanense* ACM 5017	Runoff channel formed from flowing bore water from the geothermally-heated aquifer of Great Artesian Basin, Australia	44–80°C	68°C (10 μg ml^−1^)	Andrews and Patel, 1996
*Kosmotoga pacifica* DSM 26965	Hydrothermal sediments mixed with fragments of inactive sulfide chimneys from 2891 m depth on the East Pacific Rise	33–78°C	55°C (25 μg ml^−1^)	L'Haridon et al., 2014
*Thermotoga maritima*	Sediment sample of a marine geothermal area near Vulcano, Italy	55–90°C	70°C (100 μg ml^−1^)	Huber et al., 1986
*Thermotoga petrophila* DSM 13995	Production fluid of the Kubiki oil reservoir in Niigata, Japan	47–88°C	70°C (100 μg ml^−1^)	Takahata et al., 2001
*Thermotoga naphthophila* DSM 13996	Production fluid of the Kubiki oil reservoir in Niigata, Japan	48–86°C	70°C (100 μg ml^−1^)	Takahata et al., 2001
*Petrotoga mobilis*	Anoxic samples from production water taken from the water separator tanks on off-shore oil platforms of North Sea oil reservoir	40–65°C	60°C (10 μg ml^−1^)	Lien et al., 1998
**Vancomycin response tested during chemolithotrophic growth**					
*Prote*^†^	*Thermithiobacillus tepidarius* SMMA_11	Hot water of the Lotus Pond-adjacent ebullition, Northern Puga valley, Ladakh, India	30–50°C	50°C and 30°C (50 μg ml^−1^)	This study
*Sulfurivirga caldicuralii* DSM 17737	Taketomi Island, Okinawa, Japan	30–60°C	55°C (50 μg ml^−1^)	Takai et al., 2006

*Given information were generated either in this study or collated from species descriptions reported elsewhere*.

* *Gram-variable bacteria with Gram staining response usually negative but occasionally positive*.

# Dic, Dictyoglomi;

$ Act, Actinobacteria;

† *Prote, Proteobacteria*.

### Vancomycin susceptibility of gram-negative thermophiles stems from Di-alanine-terminated MPPs

In view of the above data it was deemed imperative to know whether the typical vancomycin mode of action that works against Gram-positive bacteria (Healy et al., 2000) was also instrumental in the killing of Gram-negative thermophiles. It may be recalled that transpeptidase enzymes cross-link MPPs to provide structural integrity and strength to the peptidoglycan layer of the cell wall (Waxman and Strominger, 1983). Gram-positive bacteria generally have tandem D-ala-D-ala residues at the N-terminal end of their MPPs. These two amino acid residues afford the high-affinity target of vancomycin (Perkins and Nieto, 1974; Healy et al., 2000), which out-competes the transpeptidases in the race for binding with the substrate. In the process peptidoglycan cross-linking fails, causing the cell wall to eventually disintegrate and collapse under extraneous pressure. In contrast, a majority of Gram-negative bacteria as well as vancomycin-resistant Gram-positive bacteria have the last D-alanine replaced by D/L-lactate/lysine/glycine etc, and thus remain unscathed by vancomycin (Reynolds, 1989; Arthur et al., 1996; Courvalin, 2006). However, many others do have tandem D-ala-D-ala residues in their MPPs but impermeability of their outer membrane toward vancomycin confers them resistance to this drug. As such, the normal mechanism of vancomycin resistance starts with a permeability barrier (Shlaes et al., 1989) even as its mechanism of action is invariably rendered through binding of the terminal D-ala-D-ala residues resulting in steric hindrance of further addition to the growing peptidoglycan chain (Perkins and Nieto, 1974).

Peptidoglycan was extracted from vancomycin-treated as well as -untreated cells of the current Gram-negative thermophilic isolates and analyzed by MALDI MS. The resultant data suggested that the organisms in question were all susceptible to this antibiotic in the same mechanistic way as the Gram-positive *Firmicutes* (that is on account of having tandem alanines in the N-termini of their MPPs). As such, details of the experiment with *Thermomonas hydrothermalis* SLCR_1D is described below.

Peptidoglycan was extracted from *T*. *hydrothermalis* SLCR_1D, and analyzed in comparison with the isolates *Bacillus licheniformis* SWCR_1/2X50_9 and *A*. *kashmirensis* WT 001. Extraction was done in such a way that MPPs were represented abundantly. When the OD_600_ of a given culture reached 0.3–0.4, excess vancomycin (300 μg ml^−1^) was added to the growth media, following which they were incubated for another 4 h before cells were harvested for peptidoglycan extraction. Vancomycin-untreated cultures were passed through the same steps as above, except antibiotic addition. It was reasoned that if a test organism actually afforded the typical vancomycin target in its MPPs, this treatment would stop the cross-linking of its peptidoglycan backbone and enrich the precursors in the extract. As a final point, the extracts were digested with the muramidase, lysozyme to obtain peptidoglycan mono- or oligo-mers suitable for MALDI MS detection.

**Figure 1:**
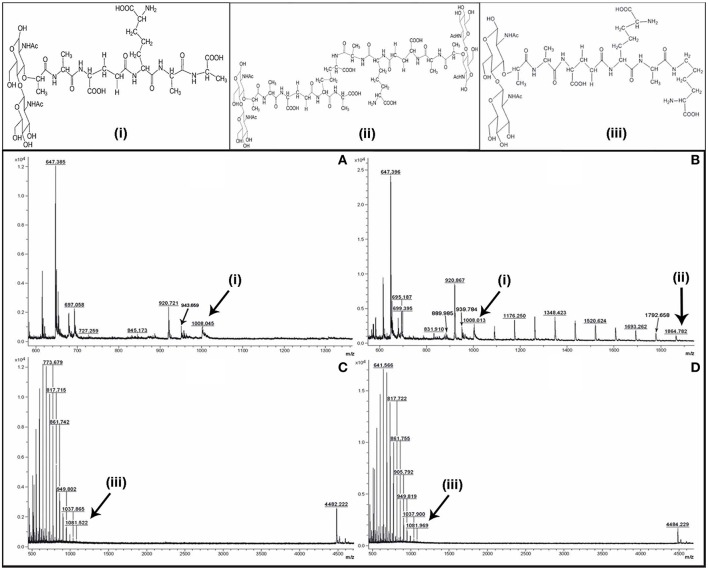
**Positive reflector ion MALDI mass spectrum of the digested peptidoglycan fragments of vancomycin-treated (A,C) and -untreated (B,D) cells of *T. hydrothermalis* SLCR_1D (Upper panels A,B) and *A. kashmirensis* WT 001 (Lower panels C,D)**. Structure **(i)** corresponds to muro-pentapeptide precursor having terminal alanine-alanine dipeptide, **(ii)** corresponds to tetrapeptide-tetrapeptide cross linking, whereas structure **(iii)** represents muro-pentapeptide precursor having terminal alanine-lysine dipeptide.

MALDI mass spectra of the SLCR_1D peptidoglycan extracted after incubation in the presence as well as absence of vancomycin encompassed a common peak (m/z ~1008) [M-3H]^+^ attributable to the monomeric precursor GlcNAc-MurNAc-ala-glu-A2pm-ala-ala (having a calculated mass average of 1011.00; Figures 1A,B). On the other hand, a unique peak of m/z 1864.782 characterized the spectrum of SLCR_1D peptidoglycan extracted after incubation without vancomycin (Figure 1B). This peak matched the calculated mass average (1861.83) of a potentially protonated [M+3H]^+^ form of the “tetrapeptide-tetrapeptide cross-linked peptidoglycan dimer.” Detectable fragmented ion peaks, or for that matter peaks arising from various modifications of the above two molecular ions of basic peptidoglycan moieties are accounted for in Table 5.

**Table 5:** **Observed and calculated average *m/z*'s along with their proposed structures and proposed modifications as present in the Figure 1**.

**Explainable peaks of Figure** **1**			**Proposed modifications**	**Presence**/**Absence in Figure** **1**			
**Obs. *m/z***	**Calculated average mass**	**Proposed structure**		**A**	**B**	**C**	**D**
1008.045 and 1008.013	1011	GlcNAc-MurNAc- Ala-Glu-A_2_Pm-Ala-Ala	-3H^+^	+	+	−	−
943.659 and 939.784	939.92	GlcNAc-MurNAc- Ala-Glu-A_2_Pm-Ala	+4H^+^	+	+	−	−
920.721 and 920.867	939.92	GlcNAc-MurNAc- Ala-Glu-A_2_Pm-Ala	-COOH+Na+H^+^	+	+	−	−
889.985	868.84	GlcNAc-MurNAc- Ala-Glu-A_2_Pm	+Na+H	+	+	−	−
845.173	868.84	GlcNAc-MurNAc- Ala-Glu-A_2_Pm	-COOH+Na+2H^+^	+	+	−	−
697	696.66	GlcNAc-MurNAc- Ala-Glu	+H^+^	+	+	−	−
1081.969 and 1081.522	1068.10	GlcNAc-MurNAc- Ala-Glu-A_2_Pm-Ala-Lys	[M+ NH${}_{4}^{+}$-3H]^+^	−	−	+	+
949.819 and 949.802	939.92	GlcNAc-MurNAc- Ala-Glu-A_2_Pm-Ala	-COOH+3NH${}_{4}^{+}$+H	−	−	+	+

+ *, presence; −, absence. All the particulars have been specified only for the monomeric fragmented ions*.

The most significant information revealed by the Figures 1A,B spectra was the presence of two tandem alanine residues at the N-terminal end of the MPP of SLCR_1D. This unambiguously accounted for the basis of vancomycin susceptibility of this Gram-negative organism. On the flip side, the absence of the “tetrapeptide-tetrapeptide cross-linked peptidoglycan dimer” in the Figure 1A confirmed that vancomycin indeed rendered total or near-total inhibition of peptidoglycan cross-linking in SLCR_1D, leading to the complete killing of this bacterium. Concurrent to these suppositions, mass spectra identical to those shown in Figures 1A,B were also obtained for *B*. *licheniformis* peptidoglycans extracted after incubation in the presence and absence of vancomycin respectively (data not shown).

Unlike *T*. *hydrothermalis, A*. *kashmirensis* yielded identical mass spectra for peptidoglycans extracted after incubation with or without vancomycin (compare Figures 1C,D). Out of the series of common peaks present in the two spectra, the one having m/z 1081.776 could be attributable [M+ NH${}_{4}^{+}$-3H]^+^ to the monomeric MPP, GlcNAc-MurNAc-ala-glu-A2pm-ala-lys, which has a calculated mass average of 1068.10. Notably, two mass peaks of m/z 4482.22 and 4484.23 present in Figures 1C,D respectively could not be explained satisfactorily. Presumably, these peaks represented some higher polymer of peptidoglycan. Fragmented ion peaks as well as peaks arising from other modifications of the aforesaid molecular ions are accounted for in Table 5.

## Concluding remarks

**Figure 2:**
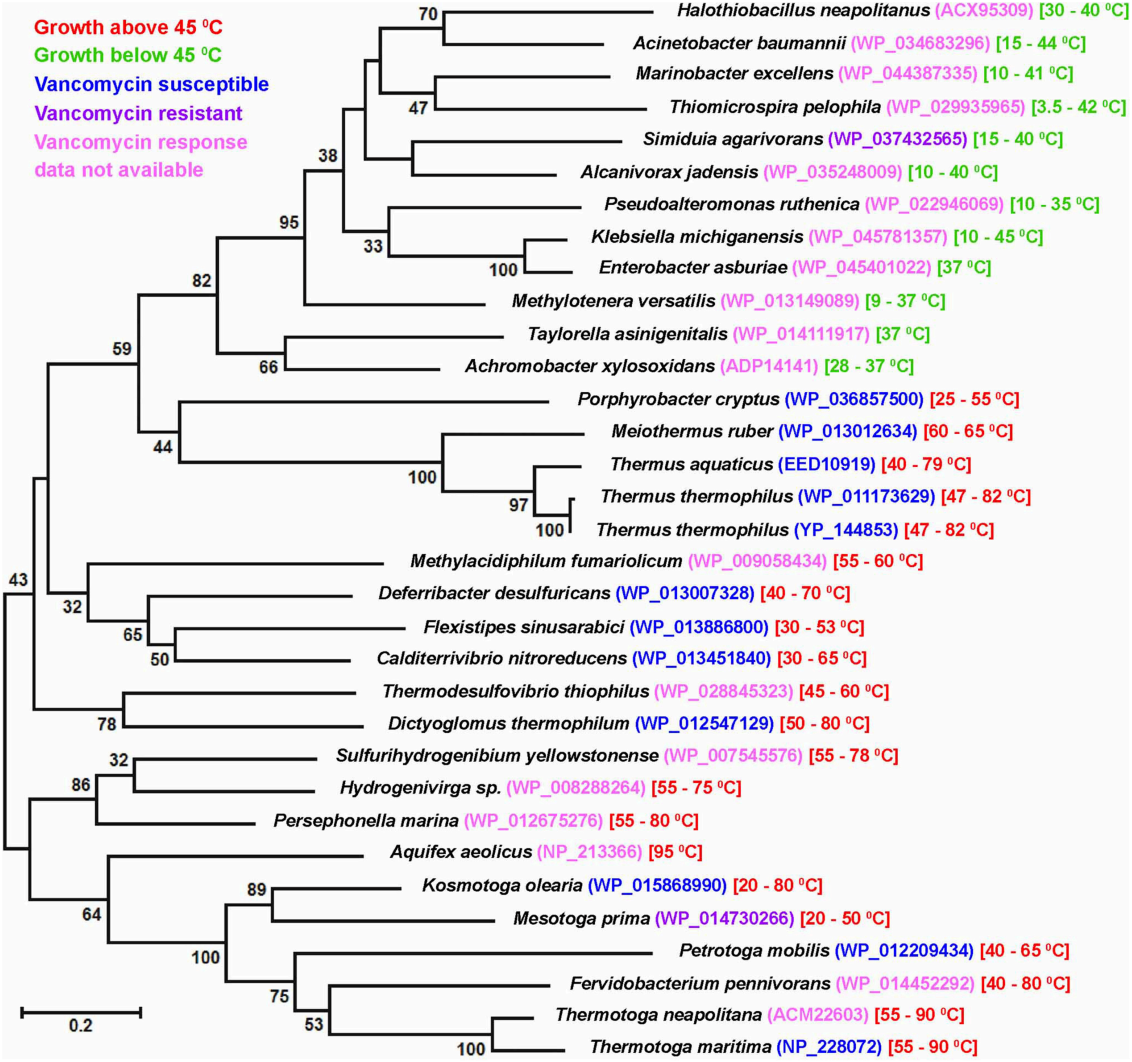
**Maximum likelihood tree constructed with D-alanine-D-alanine ligase (Ddl) sequences of Gram-negative thermophilic as well as mesophilic bacteria**. Growth temperature range of a given species is mentioned following the protein IDs of the Ddl homologs. Bootstrap support >30 (among 100 replicates) are given at the nodes. The scale bar represents two substitutions per 100 sites for a unit branch length.

All the present observations unanimously indicated that vancomycin-susceptibility was widespread in thermophilic (and perhaps also thermo-enduring) bacteria, irrespective of their taxonomic affiliation and Gram phenotype. It was also confirmed that this susceptibility stemmed from the predominance of alanine-terminated MPPs in the concerned organisms. In this regard it would be pertinent to recall that the D-ala-D-ala ligase (Ddl) of *Thermotoga maritima* (TmDdl), which can grow in the laboratory between 55 and 90°C, has been previously shown to retain its activity over a wide range of temperature (10–90°C; Sato et al., 2006). Specific-activity of this thermostable enzyme is reportedly much higher for alanine than for other amino acids (Sato et al., 2006). Interestingly, in blastp, the TmDdl (NP_228072) showed closest homology with Ddl homologs from Gram-negative thermophilic bacteria as diverse as species of *Thermotoga, Kosmotoga, Petrotoga, Mesotoga, Fervidobacterium* (phylum *Thermotogae*); *Flexistipes, Deferribacter, Calditerrivibrio* (phylum *Deferribacteres*); *Aquifex, Hydrogenivirga, Persephonella, Sulfurihydrogenibium* (phylum *Aquificae*); *Methylacidiphilum* (*Verrucomicrobia*); *Thermodesulfovibrio thiophilus* (*Nitrospirae*); *Dictyoglomus* (*Dictyoglomi*) etc. ML phylogeny not only reiterated the close affinity of these homologs but also pointed out their clear divergence from the Ddl sequences of mesophilic Gram-negative bacteria (Figure 2). Notably, among the Gram-negative thermophiles mentioned above, all the tested ones except *Mesotoga prima* have been found susceptible to vancomycin in previous studies. Since a close scrutiny of the genomes of these thermophiles revealed no second Ddl homolog it was obvious that their Ddl proteins, like the *T*. *maritima* prototype, were also adapted to favor a D-ala-D-ala bonding in MPPs, which in turn caused the handicap of vancomycin-susceptibility. In this context it was further noteworthy that the Ddl homologs of *Thermus* and *Meiothermus* species not only had lower (≤35%) identities with the TmDdl but were also widely diverged from the latter in ML phylogeny. As such, it was not surprising that previous mass spectroscopic studies had reported considerable variability in the proportion of D-ala-D-ala-terminated MPPs in *Thermus* species (Quintela et al., 1999). However, despite potential infidelity in the choice of the fifth amino acid of their MPPs, it is noteworthy that all tested *Thermales* members have till date been found susceptible to vancomycin (see Tables 3, 4). This implied that alanine was still selected as the predominant amino acid species in the fifth position of the MPPs of the *Thermales* members.

When our current findings were juxtaposed with a decades-old report showing accumulation of excess DL-alanine by thermophilic bacterial cells (Matsumoto et al., 1967) it provoked the conjecture that thermophilic processes involving freedom of amino acid choice could, in general, be skewed in favor of alanine. However, it must be acknowledged that the potential thermo-adaptive advantage (if any) conferred by D-ala-D-ala-terminated MPPs is still not clear. But, pending biophysical verification, it seems obvious that such dipeptides were preferred by bacteria in high temperature environments and vancomycin susceptibility was just a collateral consequence of this thermodynamic compulsion.

In this context it must also be appreciated that an apparently-universal preference for D-ala-D-ala-terminated MPPs still does not guarantee vancomycin susceptibility of thermophilic bacteria as a relatively-hydrophilic molecule as large as vancomycin still has to cross the outer membrane before it can inhibit peptidoglycan biosynthesis. Notably, many mesophilic Gram-negative bacteria (unlike the case of *A*. *kashmirensis* stated above) also have D-ala-D-ala-terminated MPPs, but they still remain resistant to vancomycin due to the relative impermeability of their outer membrane toward this large glycopeptide molecule (Vollmer et al., 2008; Gordon et al., 2010). As such, the above-observed vancomycin resistance of the other two Gram-negative mesophiles *Pseudaminobacter salicylatoxidans* KCT001 and *E*. *coli* BL21 could also be due to impermeability toward vancomycin. But keeping in mind the global vancomycin-susceptibility phenotype of thermophilic bacteria it is obvious that the drug manages to cross the membrane in all these cases. As a corollary of this, it also seems quite likely that the composition of the outer membranes of thermophilic bacteria have some yet-unknown characteristic feature(s) that invariably ensures the entry of such relatively-hydrophilic large molecules as vancomycin.

With regard to the apparent preference of thermophilic bacteria for D-ala-D-ala-terminated MPPs two intriguing facts remain to be clarified at length. One is the vancomycin susceptibility of the mesophilic but apparently thermo-enduring *Paracoccus* strains isolated in this study and the other is the reported vancomycin resistance of at least two Gram-negative thermophilic species in the literature (viz. *Mesotoga prima* and *Geoalkalibacter subterraneus*) (Greene et al., 2009; Nesbø et al., 2012). It is noteworthy that despite its inability to grow above 45°C, the *Paracoccus* strains were native to an ambient temperature of 70–85°C. Since such strains got frequently isolated over multiple rounds of sampling at the Lotus Pond spring system they cannot be discounted as mere stochastic introductions at the time of sampling. As such, they must be migrating intermittently from the Puga rivulet to the adjoining Lotus Pond waters and in the process getting acclimatized to elevated temperatures. In contrast, *Mesotoga prima* was isolated from an apparently mesophilic sample of unknown temperature (viz., Baltimore harbor sediment Nesbø et al., 2012), while *Geoalkalibacter subterraneus* was retrieved from formation water of an oil-well (in Redwash oilfield of Utah) that had a temperature of only 52°C (Greene et al., 2009). Additionally, *Mesotoga prima* was enriched via several months of incubation and serial sub-culturing at 22 and 30°C, whereas the current *Paracoccus* strains were isolated immediately upon retrieval of hydrothermal samples to the laboratory. In the light of the above facts vancomycin-susceptibility, and as a corollary the D-ala-D-ala-specificity of Ddl homologs, seems to be a function of thermal-conditioning of a bacterium rather than the actual upper limit of its growth temperature. As such, it would not be surprising if our *Paracoccus* isolates become vancomycin-resistant after prolonged maintenance at 28 or 37°C, or for that matter the *Mesotoga* and *Geoalkalibacter* strains become vancomycin-susceptible after several sub-cultures at ≥50°C. This kind of subtle functional switching over short evolutionary times does not seem improbable when we keep in mind the high levels of sequence divergence existing between Ddl homologs of even closely-related genera. For example, Ddl sequences of *Thermotogaceae* members *Thermotoga neapolitana* (ACM22603) and *Kosmotoga olearia* (WP_015868990) have only 43% identity, which is quite low by any “house-keeping gene” standard.

## Author contributions

CR anchored the whole work and participated in all experiments and manuscript writing. WG conceived the program, interpreted the results and wrote the manuscript. On site samplings were done by CR, MA, PH, TM, SKM, and WG. CR, MA, SM, PH, SB, TM, RR, MR, RC, and SKM did metagenomics and bioinformatics analyses. Pure culture microbiology was done by CR, MA, SM, SB, and TM. Organic chemistry experiments and data analyses were done by CR, MA, AM, RC, AN, and WG. AM, RC, AN, and SKM also contributed in improving the intellectual content of the work as well as the manuscript. All the authors read and approved the final manuscript.

### Conflict of interest statement

The authors declare that the research was conducted in the absence of any commercial or financial relationships that could be construed as a potential conflict of interest.
