# Supplementary material for: Global Association between Thermophilicity and Vancomycin Susceptibility in Bacteria
Source: Front Microbiol. 2016 Mar 31;7:412. doi: 10.3389/fmicb.2016.00412 (PMC4814524; doi:10.3389/fmicb.2016.00412)
Supplement: Supplementary file 1 [file Table1.DOC]

*Supplementary Material*

**Global association between thermophilicity and vancomycin susceptibility in bacteria**

Chayan Roy, Masrure Alam, Subhrangshu Mandal, Prabir Kumar Haldar, Sabyasachi Bhattacharya, Trinetra Mukherjee, Rimi Roy, Moidu Jameela Rameez, Anup Kumar Misra, Ranadhir Chakraborty, Ashish Kumar Nanda, Subhra Kanti Mukhopadhyay and Wriddhiman Ghosh*

*** Correspondence: Email:** [wriman@jcbose.ac.in](mailto:wriman@jcbose.ac.in)

[Wriman@rediffmail.com](mailto:Wriman@rediffmail.com)

**Phone:** 91-33-25693246

**Fax:** 91-33-23553886

**Supplementary Tables S1 through S15D**

**Table S1.** Details of OTU analysis.

| **Sample Identity** | **Total reads** | **Reads after Quality& Length filtering** | **Reads after Dereplication** | **Total OTUs**  **(minus singletons)** | **Singletons Only** | **ACE estimation**  **(minus singletons)** | **Shannon index**  **(minus singletons)** | **Simpson index**  **(minus singletons)** |
| --- | --- | --- | --- | --- | --- | --- | --- | --- |
| Lotus Pond Center | 104809 | 99654 | 30253 | 331 | 12752 | 331 | 3.1710 | 0.1340 |
| Lotus Pond-adjacent ebullition | 92201 | 84872 | 24456 | 186 | 8828 | 186 | 1.5688 | 0.4638 |
| Shivlinga | 72973 | 61342 | 14359 | 80 | 8963 | 80 | 1.3114 | 0.5270 |
| PCPR_1 | 213837 | 201983 | 15253 | 135 | 7836 | 135 | 2.6757 | 0.1337 |
| PCPR_2 | 267162 | 249453 | 44567 | 188 | 8149 | 188 | 1.6586 | 0.27911 |
| Paniphala Fountain | 737,353 | 719890 | 85672 | 743 | 6942 | 743 | 1.4547 | 0.5926 |
| Agnikunda | 996331 | 804208 | 34778 | 283 | 8237 | 283 | 0.9683 | 0.6132 |
| Kharkunda | 526337 | 426683 | 19362 | 234 | 9225 | 234 | 1.0610 | 0.5877 |
| Lotus Pond Center_R2A_30 | 57455 | 55376 | 11343 | 30 | 7899 | 30 | 0.1037 | 0.9722 |
| Lotus Pond Center_R2A_55 | 61779 | 58786 | 14598 | 58 | 9180 | 58 | 1.5276 | 0.3860 |
| Lotus Pond Center_MMST_30 | 618,398 | 602287 | 13234 | 68 | 7119 | 68 | 0.2580 | 0.9189 |
| Lotus Pond Center_MMST_55 | 980,176 | 972343 | 15623 | 136 | 9537 | 136 | 0.4964 | 0.8513 |
| Lotus Pond-adjacent ebullition_R2A_30 | 1,071,258 | 999732 | 16754 | 85 | 7906 | 85 | 0.1331 | 0.9673 |
| Lotus Pond-adjacent ebullition_R2A_55 | 1,027,829 | 972345 | 18835 | 128 | 3551 | 128 | 0.1903 | 0.9355 |
| Lotus Pond-adjacent ebullition_MMST_30 | 1,027,829 | 984697 | 12761 | 134 | 9040 | 134 | 0.4116 | 0.8543 |
| Lotus Pond-adjacent ebullition_MMST_55 | 575,974 | 550433 | 11253 | 62 | 7533 | 62 | 0.2386 | 0.9281 |
| Shivlinga_R2A_30 | 100423 | 92195 | 9877 | 54 | 6866 | 54 | 0.0953 | 0.9781 |
| Shivlinga_R2A_55 | 125414 | 112310 | 15468 | 51 | 9449 | 51 | 0.0846 | 0.9812 |
| Shivlinga_MMST_30 | 484,921 | 452984 | 35456 | 320 | 1532 | 320 | 3.7182 | 0.0495 |
| Shivlinga_MMST_55 | 602,401 | 578634 | 12398 | 51 | 8771 | 51 | 0.0926 | 0.9760 |
| Paniphala  Fountain_R2A_30 | 159435 | 145368 | 11093 | 61 | 7341 | 61 | 0.6824 | 0.7108 |
| Paniphala Fountain_R2A_55 | 197320 | 172453 | 10082 | 55 | 6982 | 55 | 0.2051 | 0.9431 |
| Paniphala  Fountain_MMST_30 | 904,251 | 885132 | 15670 | 108 | 8943 | 108 | 0.8608 | 0.4778 |
| Paniphala  Fountain_MMST_55 | 610,830 | 576930 | 13289 | 46 | 8638 | 46 | 0.1903 | 0.9400 |
| Agnikunda_R2A_30 | 267270 | 231469 | 14085 | 53 | 1957 | 53 | 0.8315 | 0.6285 |
| Agnikunda_R2A_55 | 670430 | 596672 | 26522 | 79 | 4236 | 79 | 0.1123 | 0.9694 |
| Agnikunda_MMST_30 | 758433 | 686440 | 28914 | 78 | 2076 | 78 | 0.0854 | 0.9812 |
| Agnikunda_MMST_55 | 327499 | 291301 | 12492 | 112 | 9658 | 112 | 0.2596 | 0.9324 |
| EICB_LW_1_ R2A | 975,502 | 949674 | 28954 | 310 | 9735 | 310 | 2.9903 | 0.0969 |
| EICB_LW_1_ R2A+V | 1,202,190 | 1180902 | 25487 | 226 | 6378 | 226 | 2.3906 | 0.1916 |
| EICB_LW_1_ MMST | 1,377,744 | 1322532 | 19656 | 268 | 8671 | 268 | 1.6350 | 0.4366 |
| EICB_LW_1_ MMST+V | 906,410 | 884359 | 17497 | 370 | 8305 | 370 | 1.3900 | 0.5232 |

**Table S2.** Genera identified in the V3 sequence-based OTU set of Lotus Pond Center. Temperature values above 45 ^O^C are shaded red, while those below that level are shaded green. Names of reportedly Gram negative genera are in blue font, while those of reportedly Gram positive ones are in red font. Literature search showed 23 out of the 107 (21.5%) identified genera to have reported growth above 45 ^O^C.

| **Identified genera** | **Phylum to which the**  **genera were affiliated** | **Reported optimum temperature (or temperature range) for laboratory growth (in ^O^C)** | **Reference for growth temperature(s) and Gram stain type** |
| --- | --- | --- | --- |
| *Calditerrivibrio* | Deferribacteres | 55 |  |
| *Saccharopolyspora* | Actinobacteria | 25-50 |  |
| *Ornithinimicrobium* |  | 40 |  |
| *Rothia* |  | 37 |  |
| *Brachybacterium* |  | 25-30 |  |
| *Leifsonia* |  | 24-28 |  |
| *Brevibacterium* |  | 30-37 |  |
| *Branchiibius* |  | 20–37 |  |
| *Actinomyces* |  | 37 |  |
| *Nocardioides* |  | 18-37 |  |
| *Propionibacterium* |  | 37 |  |
| *Williamsia* |  | 10-37 |  |
| *Turicella* |  | 37 |  |
| *Corynebacterium* |  | 37 |  |
| *Treponema* | Spirochaetes | 33-35 |  |
| *Soonwooa* | Bacteroidetes | 30 |  |
| *Cloacibacterium* |  | 37 |  |
| *Chryseobacterium* |  | 37 |  |
| *Psychroflexus* |  | 20 |  |
| *Prevotella* |  | 45 |  |
| *Alloprevotella* |  | 37 |  |
| *Porphyromonas* |  | 37 |  |
| *Mongoliitalea* |  | 0-40 (33) |  |
| *Sediminibacterium* |  | 18-37 |  |
| *Niabella* |  | 10-35 |  |
| *Sulfurimonas* | Proteobacteria | 10-40 |  |
| *Campylobacter* |  | 42 |  |
| *Tepidimonas* |  | 50-55 |  |
| *Aquabacterium* |  | 6-34 |  |
| *Achromobacter* |  | 41 |  |
| *Advenella* |  | 37 |  |
| *Ralstonia* |  | 41 |  |
| *Burkholderia* |  | 28 |  |
| *Duganella* |  | 28-30 |  |
| *Noviherbaspirillum* |  | 20-40 |  |
| *Herbaspirillum* |  | 35 |  |
| *Acidovorax* |  | 37 |  |
| *Pelomonas* |  | 4-40 |  |
| *Methylophilus* |  | 37 |  |
| *Vogesella* |  | 40 |  |
| *Methyloversatilis* |  | 30-37 |  |
| *Azovibrio* |  | 40 |  |
| *Bradyrhizobium* |  | 30 |  |
| *Methylobacterium* |  | 30-37 |  |
| *Brevundimonas* |  | 30-37 |  |
| *Sphingomonas* |  | 40 |  |
| *Azospirillum* |  | 30 |  |
| *Paracoccus* |  | 8-40 |  |
| *Sulfitobacter* |  | 4-35 |  |
| *Acinetobacter* |  | 41 |  |
| *Enhydrobacter* |  | 20-41 |  |
| *Psychrobacter* |  | 20-25 |  |
| *Thiofaba* |  | 20-51 |  |
| *Thiovirga* |  | 30-34 (40) |  |
| *Alcanivorax* |  | 4-35 |  |
| *Salinicola* |  | 30-37 |  |
| *Halomonas* |  | 4-45 (37) |  |
| *Chromohalobacter* |  | 5-45 (37) |  |
| *Providencia* |  | 37 |  |
| *Serratia* |  | 60 |  |
| *Haemophilus* |  | 20-40 (37) |  |
| *Nevskia* |  | 20-25 |  |
| *Lysobacter* |  | 25-40 |  |
| *Thermomonas* |  | 18-50 |  |
| *Stenotrophomonas* |  | 35 |  |
| *Photobacterium* |  | 4-18 |  |
| *Aeromonas* |  | 41 |  |
| *Colwellia* |  | 15 |  |
| *Marinimicrobium* |  | 10-45 |  |
| *Marinobacter* |  | 10-45 |  |
| *Shewanella* |  | 30 |  |
| *Thiothrix* |  | 25-30 |  |
| *Methylophaga* |  | 10-40 |  |
| *Thiomicrospira* |  | 37 |  |
| *Thermotoga* | Thermotogae | 55-90 |  |
| *Fervidobacterium* |  | 40-80 |  |
| *Geothrix* |  | 35 |  |
| *Sulfurihydrogenibium* | Aquificae | 40-70 |  |
| *Hydrogenobacter* |  | 75 |  |
| *Clostridium* | Firmicutes | 65-68 |  |
| *Peptoniphilus* |  | 37 |  |
| *Anaerococcus* |  | 37 |  |
| *Caldicellulosiruptor* |  | 50-78 |  |
| *Streptococcus* |  | 37 |  |
| *Lactococcus* |  | 43 |  |
| *Enterococcus* |  | 10-45 |  |
| *Lactobacillus* |  | 32 (>15 &<45) |  |
| *Domibacillus* |  | 35 |  |
| *Geobacillus* |  | 37-65 |  |
| *Anoxybacillus* |  | 37-66 |  |
| *Bacillus* |  | 5-55 |  |
| *Brevibacillus* |  | 50 |  |
| *Paenibacillus* |  | 23-37 |  |
| *Exiguobacterium* |  | 7-43 |  |
| *Planococcus* |  | 20-37 |  |
| *Chryseomicrobium* |  | 4-45 |  |
| *Salinicoccus* |  | 15-37 |  |
| *Macrococcus* |  | 35 |  |
| *Staphylococcus* |  | 30-40 |  |
| *Turicibacter* |  | 25-46 |  |
| *Deinococcus* | Deinococcus-Thermus | 30 |  |
| *Truepera (Indeterminate)* |  | 25-55 (50) |  |
| *Meiothermus* |  | 60-65 |  |
| *Thermus* |  | 70-75 |  |
| *Ignavibacterium* | Ignavibacteriae | 30-55 (45) |  |
| *Roseiflexus* | Chloroflexi | 40-60 (50) |  |
| *Chloroflexus* |  | 52-60 |  |

**Table S3.** Genera identified in the V3 sequence-based OTU set of Lotus Pond-adjacent ebullition.Temperature values above 45 ^O^C are shaded red, while those below that level are shaded green. Names of reportedly Gram negative genera are in blue font, while those of reportedly Gram positive ones are in red font.Literature search showed 5 out of the 28 (17.9%) identified genera to have reported growth above 45 ^O^C.

| **Identified genera** | **Phylum to which the**  **genera were affiliated** | **Reported optimum temperature (or temperature range) for laboratory growth (in ^O^C)** | **Reference for growth temperature(s) and Gram stain type** |
| --- | --- | --- | --- |
| *Calditerrivibrio* | Deferribacteres | 55 |  |
| *Ilumatobacter* | Actinobacteria | 26-31 |  |
| *Nitriliruptor* |  | 32 |  |
| *Ornithinimicrobium* |  | 40 |  |
| *Demequina* |  | 5-35 |  |
| *Cryobacterium* |  | 9-12 |  |
| *Actinomyces* |  | 37 |  |
| *Nocardioides* |  | 18-37 |  |
| *Friedmanniella* |  | 9-25 |  |
| *Propionibacterium* |  | 37 |  |
| *Corynebacterium* |  | 37 |  |
| *Treponema* | Spirochaetes | 33-35 |  |
| *Leptospira* |  | 28-30 |  |
| *Gillisia* | Bacteroidetes | 5-30 |  |
| *Flavobacterium* |  | 0-42 |  |
| *Cloacibacterium* |  | 37 |  |
| *Lutaonel* |  | 25-55 |  |
| *Weissella* | Firmicutes | 40 |  |
| *Alkalibacterium* |  | 27-32 |  |
| *Lactobacillus* |  | 32 (>15 &<45) |  |
| *Geobacillus* |  | 37-65 |  |
| *Anoxybacillus* |  | 37-66 |  |
| *Bacillus* |  | 5-55 |  |
| *Terribacillus* |  | 5-45 |  |
| *Marinococcus* |  | 30-37 |  |
| *Brevibacillus* |  | 50 |  |
| *Paenibacillus* |  | 23-37 |  |
| *Exiguobacterium* |  | 7-43 |  |

**Table S4.** Genera identified in the V3 sequence-based OTU set of Shivlinga.Temperature values above 45 ^O^C are shaded red, while those below that level are shaded green. Names of reportedly Gram negative genera are in blue font, while those of reportedly Gram positive ones are in red font.Literature search showed 10 out of the 25 (40%) identified genera to have reported growth above 45 ^O^C.

| **Identified genera** | **Phylum to which the**  **genera were affiliated** | **Reported optimum temperature (or temperature range) for laboratory growth (in ^O^C)** | **Reference for growth temperature(s) and Gram stain type** |
| --- | --- | --- | --- |
| *Calditerrivibrio* | Deferribacteres | 55 |  |
| *Brachybacterium* | Actinobacteria | 25-30 |  |
| *Propionibacterium* |  | 37 |  |
| *Myroides* | Bacteroidetes | 18-37 |  |
| *Advenella* | Proteobacteria | 37 |  |
| *Burkholderia* |  | 28 |  |
| *Methylobacterium* |  | 30-37 |  |
| *Paracoccus* |  | 8-40 |  |
| *Acinetobacter* |  | 41 |  |
| *Thiofaba* |  | 20-51 |  |
| *Halomonas* |  | 4-45 (37) |  |
| *Thermotoga* | Thermotogae | 55-90 |  |
| *Fervidobacterium* |  | 40-80 |  |
| *Sulfurihydrogenibium* | Aquificae | 40-70 |  |
| *Streptococcus* | Firmicutes | 37 |  |
| *Anoxybacillus* |  | 37-66 |  |
| *Bacillus* |  | 5-55 |  |
| *Brevibacillus* |  | 50 |  |
| *Paenibacillus* |  | 23-37 |  |
| *Exiguobacterium* |  | 7-43 |  |
| *Planococcus* |  | 20-37 |  |
| *Planomicrobium* |  | 4-38 |  |
| *Staphylococcus* |  | 30-40 |  |
| *Thermus* | Deinococcus-Thermus | 70-75 |  |
| *Thermodesulfovibrio* | Nitrospirae | 40-70 |  |

**Table S5.** Genera identified in the V3 sequence-based OTU set of PCPR_1.Temperature values above 45 ^O^C are shaded red, while those below that level are shaded green. Names of reportedly Gram negative genera are in blue font, while those of reportedly Gram positive ones are in red font. Literature search showed 9 out of the 20 (45%) identified genera to have reported growth above 45 ^O^C.

| ***Identified genera*** | **Phylum to which the**  **genera were affiliated** | **Reported optimum temperature (or temperature range) for laboratory growth (in ^O^C)** | **Reference for growth temperature(s) and Gram stain type** |
| --- | --- | --- | --- |
| *Nitriliruptor* | Actinobacteria | 32 |  |
| *Agromyces* |  | 30 |  |
| *Tepidimonas* | Proteobacteria | 50-55 |  |
| *Advenella* |  | 37 |  |
| *Hyphomicrobium* |  | 30 |  |
| *Brevundimonas* |  | 30-37 |  |
| *Elioraea* |  | 45-50 |  |
| *Paracoccus* |  | 8-40 |  |
| *Rubellimicrobium* |  | 45-54 |  |
| *Acinetobacter* |  | 41 |  |
| *Silanimonas* |  | 25-53 |  |
| *Lysobacter* |  | 25-40 |  |
| *Thermomonas* |  | 18-50 |  |
| *Fervidobacterium* | Thermotogae | 40-80 |  |
| *Sulfurihydrogenibium* | Aquificae | 40-70 |  |
| *Aerococcus* | Firmicutes | 22-37 |  |
| *Bacillus* |  | 5-55 |  |
| *Paenibacillus* |  | 23-37 |  |
| *Lysinibacillus* |  | 16-45 |  |
| *Ignavibacterium* | Ignavibacteriae | 30-55 |  |

**Table S6.**Genera identified in the V3 sequence-based OTU set of PCPR_2.Temperature values above 45 ^O^C are shaded red, while those below that level are shaded green. Names of reportedly Gram negative genera are in blue font, while those of reportedly Gram positive ones are in red font. Literature search showed 9 out of the 22 (41%) identified genera to have reported growth above 45 ^O^C.

| **Identified genera** | **Phylum to which the**  **genera were affiliated** | **Reported optimum temperature (or temperature range) for laboratory growth (in ^O^C)** | **Reference for growth temperature(s) and Gram stain type** |
| --- | --- | --- | --- |
| *Agromyces* | Actinobacteria | 30 |  |
| *Turneriella* | Spirochaetes | 37 |  |
| *Advenella* | Proteobacteria | 37 |  |
| *Burkholderia* |  | 28 |  |
| *Variovorax* |  | 28-30 |  |
| *Methylophilus* |  | 37 |  |
| *Bradyrhizobium* |  | 30 |  |
| *Brevundimonas* |  | 30-37 |  |
| *Elioraea* |  | 45-50 |  |
| *Roseomonas* |  | 25-42 |  |
| *Rhodobacter* |  | 30 |  |
| *Paracoccus* |  | 8-40 |  |
| *Acinetobacter* |  | 41 |  |
| *Halomonas* |  | 4-45 (37) |  |
| *Silanimonas* |  | 25-53 |  |
| *Bacillus* | Firmicutes | 5-55 |  |
| *Brevibacillus* |  | 50 |  |
| *Ignavibacterium* | Ignavibacteriae | 30-55 |  |
| *Bellilinea* | Chloroflexi | 45-65 |  |
| *Caldilinea* |  | 37-65 |  |
| *Roseiflexus* |  | 40-60 |  |
| *Chloroflexus* |  | 52-60 |  |

**Table S7.** Genera identified in the V3 sequence-based OTU set of Paniphala Fountain. Temperature values above 45 ^O^C are shaded red, while those below that level are shaded green. Names of reportedly Gram negative genera are in blue font, while those of reportedly Gram positive ones are in red font. One organism, viz. *Jeotgalibacillus*, which was reported to be “Gram variable”, is written in black font. Literature search showed 10 out of the 91 (11%) identified genera to have reported growth above 45 ^O^C.

| **Identified genera** | **Phylum to which the**  **genera were affiliated** | **Reported optimum temperature (or temperature range) for laboratory growth (in ^O^C)** | **Reference for growth temperature(s) and Gram stain type** |
| --- | --- | --- | --- |
| *Leptotrichia* | Fusobacteria | 25-42 |  |
| *Nitriliruptor* | Actinobacteria | 32 |  |
| *Prauserella* |  | 10-45 |  |
| *Actinomycetospora* |  | 28 |  |
| *Pseudonocardia* |  | 40-45 |  |
| *Ornithinimicrobium* |  | 40 |  |
| *Rothia* |  | 37 |  |
| *Piscicoccus* |  | 10-37 |  |
| *Brachybacterium* |  | 25-30 |  |
| *Agromyces* |  | 30 |  |
| *Leifsonia* |  | 24-28 |  |
| *Brevibacterium* |  | 30-37 |  |
| *Nocardioides* |  | 18-37 |  |
| *Propionibacterium* |  | 37 |  |
| *Dietzia* |  | 7-45 |  |
| *Corynebacterium* |  | 37 |  |
| *Planobacterium* | Bacteroidetes | 18-37 |  |
| *Zunongwangia* |  | 4-38 |  |
| *Chryseobacterium* |  | 37 |  |
| *Elizabethkingia* |  | 28-37 |  |
| *Maribacter* |  | 4-33 |  |
| *Adhaeribacter* |  | 4-37 |  |
| *Flectobacillus* |  | 4-30 |  |
| *Prevotella* |  | 45 |  |
| *Porphyromonas* |  | 37 |  |
| *Sphingobacterium* |  | 5-40 |  |
| *Sediminibacterium* |  | 18-37 |  |
| *Arcobacter* | Proteobacteria | 15-37 |  |
| *Aquabacterium* |  | 6-34 |  |
| *Achromobacter* |  | 41 |  |
| *Advenella* |  | 37 |  |
| *Ralstonia* |  | 41 |  |
| *Polynucleobacter* |  | 5-35 |  |
| *Burkholderia* |  | 28 |  |
| *Duganella* |  | 28-30 |  |
| *Naxibacter* |  | 4-55 |  |
| *Comamonas* |  | 37 |  |
| *Delftia* |  | 5-40 |  |
| *Pelomonas* |  | 4-40 |  |
| *Methylobacillus* |  | 20-30 |  |
| *Methylophilus* |  | 37 |  |
| *Deefgea* |  | 4-32 |  |
| *Uruburuella* |  | 37 |  |
| *Ancylobacter* |  | 30 |  |
| *Pedomicrobium* |  | 10-40 |  |
| *Methylobacterium* |  | 30-37 |  |
| *Brevundimonas* |  | 30-37 |  |
| *Sphingomonas* |  | 40 |  |
| *Azospirillum* |  | 30 |  |
| *Acetobacter* |  | 25-40 |  |
| *Paracoccus* |  | 8-40 |  |
| *Acinetobacter* |  | 41 |  |
| *Enhydrobacter* |  | 20-41 |  |
| *Psychrobacter* |  | 20-25 |  |
| *Rheinheimera* |  | 4-30 |  |
| *Halomonas* |  | 4-45 (37) |  |
| *Chromohalobacter* |  | 5-45 (37) |  |
| *Providencia* |  | 37 |  |
| *Serratia* |  | 60 |  |
| *Thermomonas* |  | 18-50 |  |
| *Salinivibrio* |  | 5-45 |  |
| *Lucibacterium* |  | 37 |  |
| *Aeromonas* |  | 41 |  |
| *Aliidiomarina* |  | 4-45 |  |
| *Alishewanella* |  | 25-42 |  |
| *Shewanella* |  | 30 |  |
| *Anaerococcus* | Firmicutes | 37 |  |
| *Butyricicoccus* |  | 42 |  |
| *Clostridium* |  | 65-68 |  |
| *Streptococcus* |  | 37 |  |
| *Lactococcus* |  | 43 |  |
| *Enterococcus* |  | 10-45 |  |
| *Aerococcus* |  | 22-37 |  |
| *Weissella* |  | 40 |  |
| *Alkalibacterium* |  | 27-32 |  |
| *Lactobacillus* |  | 32 (>15 &<45) |  |
| *Geobacillus* |  | 37-65 |  |
| *Anoxybacillus* |  | 37-66 |  |
| *Bacillus* |  | 5-55 |  |
| *Terribacillus* |  | 5-45 |  |
| *Marinococcus* |  | 30-37 |  |
| *Brevibacillus* |  | 50 |  |
| *Paenibacillus* |  | 23-37 |  |
| *Exiguobacterium* |  | 7-43 |  |
| *Jeotgalibacillus* |  | 10-45 |  |
| *Planococcus* |  | 20-37 |  |
| *Chryseomicrobium* |  | 4-45 |  |
| *Staphylococcus* |  | 30-40 |  |
| *Deinococcus* | Deinococcus-Thermus | 30 |  |
| *Meiothermus* |  | 60-65 |  |
| *Thermodesulfovibrio* | Nitrospirae | 40-70 |  |

| **Identifiedgenera** | **Phylum to which the**  **genera were affiliated** | **Reported optimum temperature (or temperature range) for laboratory growth (in ^O^C)** | **Reference for growth temperature(s) and Gram stain type** |
| --- | --- | --- | --- |
| *Saccharopolyspora* | Actinobacteria | 25-50 |  |
| *Ornithinimicrobium* |  | 40 |  |
| *Microbacterium* |  | 15-42 |  |
| *Brevibacterium* |  | 30-37 |  |
| *Propionibacterium* |  | 37 |  |
| *Flavobacterium* | Bacteroidetes | 0-42 |  |
| *Sediminibacterium* |  | 18-37 |  |
| *Advenella* | Proteobacteria | 37 |  |
| *Burkholderia* |  | 28 |  |
| *Bosea* |  | 20-37 |  |
| *Ancylobacter* |  | 30 |  |
| *Methylobacterium* |  | 30-37 |  |
| *Brevundimonas* |  | 30-37 |  |
| *Sphingomonas* |  | 40 |  |
| *Paracoccus* |  | 8-40 |  |
| *Acinetobacter* |  | 41 |  |
| *Enhydrobacter* |  | 20-41 |  |
| *Pseudomonas* |  | 10-42 |  |
| *Halomonas* |  | 4-45 (37) |  |
| *Serratia* |  | 60 |  |
| *Thermotoga* | Thermotogae | 55-90 |  |
| *Fervidobacterium* |  | 40-80 |  |
| *Hydrogenobacter* | Aquificae | 75 |  |
| *Caloramator* | Firmicutes | 37-80 |  |
| *Thermosyntropha* |  | 52-70 |  |
| *Thermovenabulum* |  | 45-76 |  |
| *Caldicellulosiruptor* |  | 50-78 |  |
| *Clostridium* |  | 65-68 |  |
| *Thermoanaerobacter* |  | 37-78 |  |
| *Weissella* |  | 40 |  |
| *Geobacillus* |  | 37-65 |  |
| *Anoxybacillus* |  | 37-66 |  |
| *Bacillus* |  | 5-55 |  |
| *Brevibacillus* |  | 50 |  |
| *Chryseomicrobium* |  | 4-45 |  |
| *Lysinibacillus* |  | 16-45 |  |
| *Salinicoccus* |  | 15-37 |  |
| *Staphylococcus* |  | 30-40 |  |
| *Sporolituus* |  | 45-60 |  |
| *Deinococcus* | Deinococcus-Thermus | 30 |  |
| *Meiothermus* |  | 60-65 |  |
| *Thermus* |  | 70-75 |  |
| *Thermodesulfovibrio* | Nitrospirae | 40-70 |  |
| *Dictyoglomus* | Dictyoglomi | 50-80 |  |
| *Caldilinea* | Chloroflexi | 37-65 |  |
| *Chloroflexus* |  | 52-60 |  |

**Table S8.** Genera identified in the V3 sequence-based OTU set of Agnikunda. Temperature values above 45 ^O^C are shaded red, while those below that level are shaded green. Names of reportedly Gram negative genera are in blue font, while those of reportedly Gram positive ones are in red font. One organism, viz. *Thermoanaerobacter*, which was reported to be “Gram-variable”, is written in black font. Literature search showed 22 out of the 46 (47.82%) identified genera to have reported growth above 45 ^O^C.

**Table S9.** Genera identified in the V3 sequence-based OTU set of Kharkunda. Temperature values above 45 ^O^C are shaded red, while those below that level are shaded green. Names of reportedly Gram negative genera are in blue font, while those of reportedly Gram positive ones are in red font. One organism, viz. *Thermoanaerobacter*, which was reported to be Gram-variable, is written in black font. Literature search showed 23 out of the 58 (39.65%) identified genera to have reported growth above 45 ^O^C.

| **Identifiedgenera** | **Phylum to which the**  **genera were affiliated** | **Reported optimum temperature (or temperature range) for laboratory growth (in ^O^C)** | **Reference for growth temperature(s) and Gram stain type** |
| --- | --- | --- | --- |
| *Saccharopolyspora* | Actinobacteria | 25-50 |  |
| *Brachybacterium* |  | 25-30 |  |
| *Microbacterium* |  | 15-42 |  |
| *Brevibacterium* |  | 30-37 |  |
| *Nocardioides* |  | 18-37 |  |
| *Propionibacterium* |  | 37 |  |
| *Cloacibacterium* | Bacteroidetes | 37 |  |
| *Chryseobacterium* |  | 37 |  |
| *Advenella* | Proteobacteria | 37 |  |
| *Burkholderia* |  | 28 |  |
| *Delftia* |  | 5-40 |  |
| *Hydrogenophaga* |  | 30-37 |  |
| *Methylophilus* |  | 37 |  |
| *Rhodoblastus* |  | 25-30 |  |
| *Kaistia* |  | 10-37 |  |
| *Aurantimonas* |  | 28-37 |  |
| *Methylobacterium* |  | 30-37 |  |
| *Brevundimonas* |  | 30-37 |  |
| *Sphingomonas* |  | 40 |  |
| *Roseomonas* |  | 25-42 |  |
| *Paracoccus* |  | 8-40 |  |
| *Rubellimicrobium* |  | 45-54 |  |
| *Acinetobacter* |  | 41 |  |
| *Enhydrobacter* |  | 20-41 |  |
| *Pseudomonas* |  | 10-42 |  |
| *Halomonas* |  | 4-45 (37) |  |
| *Hydrocarboniphaga* |  | 28 |  |
| *Stenotrophomonas* |  | 35 |  |
| *Methylothermus* |  | 37–67 |  |
| *Thermotoga* | Thermotogae | 55-90 |  |
| *Fervidobacterium* |  | 40-80 |  |
| *Caloramator* | Firmicutes | 37-80 |  |
| *Clostridium* |  | 65-68 |  |
| *Thermosyntropha* |  | 52-70 |  |
| *Thermovenabulum* |  | 45-76 |  |
| *Caldicellulosiruptor* |  | 50-78 |  |
| *Caldicoprobacter* |  | 44-77 |  |
| *Thermoanaerobacter* |  | 37-78 |  |
| *Streptococcus* |  | 37 |  |
| *Lactobacillus* |  | 15-45 |  |
| *Geobacillus* |  | 37-65 |  |
| *Anoxybacillus* |  | 37-66 |  |
| *Bacillus* |  | 5-55 |  |
| *Brevibacillus* |  | 50 |  |
| *Exiguobacterium* |  | 7-43 |  |
| *Tumebacillus* |  | 5–37 |  |
| *Kurthia* |  | 30 |  |
| *Chryseomicrobium* |  | 4-45 |  |
| *Lysinibacillus* |  | 16-45 |  |
| *Macrococcus* |  | 35 |  |
| *Staphylococcus* |  | 30-40 |  |
| *Thermoactinomyces* |  | 55 |  |
| *Sporolituus* |  | 45-60 |  |
| *Meiothermus* | Deinococcus-Thermus | 60-65 |  |
| *Thermus* |  | 70-75 |  |
| *Thermodesulfovibrio* | Nitrospirae | 40-70 |  |
| *Dictyoglomus* | Dictyoglomi | 50-80 |  |
| *Chloroflexus* | Chloroflexi | 52-60 |  |

**Table S10A.** Genera identified after incubating the Lotus Pond Center inoculum in R2A medium at 30 ^O^C. The cultured metagenome was prepared after 16 hours of incubation when OD_600_ of the spent medium was 0.8. V3 regions of all bacterial 16S rRNA genes were PCR-amplified and sequenced on an Ion PGM.

| **Genus** | **Phylum** | **Gram Stain of the type strain** | **Reference** |
| --- | --- | --- | --- |
| *Bacillus* | Firmicutes | Positive |  |

**Table S10B. Genera identified after incubating the Lotus Pond Center inoculum in R2A medium at 55 ^O^C. The cultured metagenome was prepared after 12 hours of incubation when OD_600_ of the spent medium was 0.8. V3regions of all bacterial 16S rRNA genes were PCR-amplified and sequenced on an Ion PGM.**

| **Genus** | **Phylum** | **Gram Stain of the type strain** | **Reference** |
| --- | --- | --- | --- |
| *Thermoleophilum* | Actinobacteria | Negative |  |
| *Ornithinimicrobium* |  | Positive |  |
| *Propionibacterium* |  | Positive |  |
| *Streptomyces* |  | Positive |  |
| *Advenella* | Proteobacteria | Negative |  |
| *Burkholderia* |  | Negative |  |
| *Variovorax* |  | Negative |  |
| *Brevundimonas* |  | Negative |  |
| *Acinetobacter* |  | Negative |  |
| *Enhydrobacter* |  | Negative |  |
| *Pseudomonas* |  | Negative |  |
| *Providencia* |  | Negative |  |
| *Thermomonas* |  | Negative |  |
| *Vibrio* |  | Negative |  |
| *Geobacillus* | Firmicutes | Positive |  |
| *Bacillus* |  | Positive |  |
| *Brevibacillus* |  | Positive |  |
| *Staphylococcus* |  | Positive |  |

**Table S10C. Genera identified after incubating the Lotus Pond Center inoculum in MMST medium at 30 ^O^C. The cultured metagenome was prepared after 72 hours of incubation when OD_600_ of the spent medium was 0.3 and pH 6.0. V3 regions of all bacterial 16S rRNA genes were PCR-amplified and sequenced on an Ion PGM.**

| **Genus** | **Phylum** | **Gram Stain of the type strain** | **Reference** |
| --- | --- | --- | --- |
| *Methylobacterium* | Proteobacteria | Negative |  |
| *Geobacillus* | Firmicutes | Positive |  |
| *Anoxybacillus* |  | Positive |  |

**Table S10D. Genera identified after incubating the Lotus Pond Center inoculum in MMST medium at 55 ^O^C. The cultured metagenome was prepared after 36 hours of incubation when OD_600_ of the spent medium was 0.4 and pH 5.0. V3 regions of all bacterial 16S rRNA genes were PCR-amplified and sequenced on an Ion PGM.**

| **Genus** | **Phylum** | **Gram Stain of the type strain** | **Reference** |
| --- | --- | --- | --- |
| *Propionibacterium* | Actinobacteria | Positive |  |
| *Sediminibacterium* | Bacteroidetes | Negative |  |
| *Advenella* | Proteobacteria | Negative |  |
| *Burkholderia* |  | Negative |  |
| *Methylobacterium* |  | Negative |  |
| *Brevundimonas* |  | Negative |  |
| *Sphingomonas* |  | Negative |  |
| *Rhodobacter* |  | Negative |  |
| *Paracoccus* |  | Negative |  |
| *Sulfitobacter* |  | Negative |  |
| *Acinetobacter* |  | Negative |  |
| *Pseudomonas* |  | Negative |  |
| *Halomonas* |  | Negative |  |
| *Sulfurihydrogenibium* | Aquificae | Negative |  |
| *Streptococcus* | Firmicutes | Positive |  |
| *Geobacillus* |  | Positive |  |
| *Anoxybacillus* |  | Positive |  |

**Table S11A.** Genera identified after incubating the Lotus Pond-adjacent ebullition inoculum in R2A medium at 30 ^O^C. The cultured metagenome was prepared after 16 hours of incubation when OD_600_ of the spent medium was 0.8. V3 regions of all bacterial 16S rRNA genes were PCR-amplified and sequenced on an Ion PGM.

| **Genus** | **Phylum** | **Gram Stain of the type strain** | **Reference** |
| --- | --- | --- | --- |
| *Thiothrix* | Proteobacteria | Negative |  |
| *Streptococcus* | Firmicutes | Positive |  |
| *Enterococcus* |  | Positive |  |
| *Bacillus* |  | Positive |  |
| *Brevibacillus* |  | Positive |  |
| *Lysinibacillus* |  | Positive |  |

**Table S11B.** Genera identified after incubating the Lotus Pond-adjacent ebullition inoculum in R2A medium at 55 ^O^C. The cultured metagenome was prepared after 12 hours of incubation when OD_600_ of the spent medium was 0.8. V3 regions of all bacterial 16S rRNA genes were PCR-amplified and sequenced on an Ion PGM.

| **Genus** | **Phylum** | **Gram Stain of the type strain** | **Reference** |
| --- | --- | --- | --- |
| *Rhizobium* | Proteobacteria | Negative |  |
| *Thiovirga* |  | Negative |  |
| *Enterococcus* | Firmicutes | Positive |  |
| *Anoxybacillus* |  | Positive |  |
| *Bacillus* |  | Positive |  |
| *Brevibacillus* |  | Positive |  |
| *Rhodococcus* | Actinobacteria | Positive |  |

**Table S11C. Genera identified after incubating the Lotus Pond-adjacent ebullition inoculum in MMST medium at 30 ^O^C. The cultured metagenome was prepared after 48 hours of incubation when OD_600_ of the spent medium was 0.3 and pH 5.3. V3 regions of all bacterial 16S rRNA genes were PCR-amplified and sequenced on an Ion PGM.**

| **Genus** | **Phylum** | **Gram Stain of the type strain** | **Reference** |
| --- | --- | --- | --- |
| *Pseudomonas* | Proteobacteria | Negative |  |
| *Bacillus* | Firmicutes | Positive |  |
| *Brevibacillus* |  | Positive |  |
| *Exiguobacterium* |  | Positive |  |
| *Lysinibacillus* |  | Positive |  |

**Table S11D.** Genera identified after incubating the Lotus Pond-adjacent ebullition inoculum in MMST medium at 55 ^O^C. The cultured metagenome was prepared after 36 hours of incubation when OD_600_ of the spent medium was 0.3 and pH 5.5. V3 regions of all bacterial 16S rRNA genes were PCR-amplified and sequenced on an Ion PGM.

| **Genus** | **Phylum** | **Gram Stain of the type strain** | **Reference** |
| --- | --- | --- | --- |
| *Pseudonocardia* | Actinobacteria | Positive |  |
| *Advenella* | Proteobacteria | Negative |  |
| *Burkholderia* |  | Negative |  |
| *Methylobacterium* |  | Negative |  |
| *Halomonas* |  | Negative |  |
| *Anoxybacillus* | Firmicutes | Positive |  |
| *Bacillus* |  | Positive |  |
| *Brevibacillus* |  | Positive |  |
| *Exiguobacterium* |  | Positive |  |
| *Lysinibacillus* |  | Positive |  |
| *Staphylococcus* |  | Positive |  |

**Table S12A.** Genera identified after incubating the Shivlinga inoculum in R2A medium at 30 ^O^C. The cultured metagenome was prepared after 16 hours of incubation when OD_600_ of the spent medium was 0.8. V3 regions of all bacterial 16S rRNA genes were PCR-amplified and sequenced on an Ion PGM.

| **Genus** | **Phylum** | **Gram Stain of the type strain** | **Reference** |
| --- | --- | --- | --- |
| *Pseudonocardia* | Actinobacteria | Positive |  |
| *Propionibacterium* |  | Positive |  |
| *Advenella* | Proteobacteria | Negative |  |
| *Burkholderia* |  | Negative |  |
| *Methylophilus* |  | Negative |  |
| *Methylobacterium* |  | Negative |  |
| *Brevundimonas* |  | Negative |  |
| *Enhydrobacter* |  | Negative |  |
| *Pseudomonas* |  | Negative |  |
| *Thiofaba* |  | Negative |  |
| *Thermomonas* |  | Negative |  |
| *Sulfurihydrogenibium* | Aquificae | Negative |  |
| *Anoxybacillus* | Firmicutes | Positive |  |
| *Bacillus* |  | Positive |  |
| *Brevibacillus* |  | Positive |  |
| *Thermus* | Deinococcus-Thermus | Negative |  |

**Table S12B.** Genera identified after incubating the Shivlinga inoculum in R2A medium at 55 ^O^C. The cultured metagenome was prepared after 12 hours of incubation when OD_600_ of the spent medium was 0.8. V3 regions of all bacterial 16S rRNA genes were PCR-amplified and sequenced on an Ion PGM.

| **Genus** | **Phylum** | **Gram Stain of the type strain** | **Reference** |
| --- | --- | --- | --- |
| *Propionibacterium* | Actinobacteria | Positive |  |
| *Advenella* | Proteobacteria | Negative |  |
| *Pseudomonas* |  | Negative |  |
| *Sulfurihydrogenibium* | Aquificae | Negative |  |
| *Geobacillus* | Firmicutes | Positive |  |
| *Thermus* | Deinococcus-Thermus | Negative |  |
| *Caldilinea* | Chloroflexi | Negative |  |

**Table S12C. Genera identified after incubating the Shivlinga inoculum in MMST medium at 30 ^O^C. The cultured metagenome was prepared after 36 hours of incubation when OD_600_ of the spent medium was 0.3 and pH 6.0. V3 regions of all bacterial 16S rRNA genes were PCR-amplified and sequenced on an Ion PGM.**

| **Genus** | **Phylum** | **Gram Stain of the type strain** | **Reference** |
| --- | --- | --- | --- |
| *Nitriliruptor* | Actinobacteria | Positive |  |
| *Rubrobacter* |  | Positive |  |
| *Pseudonocardia* |  | Positive |  |
| *Saccharopolyspora* |  | Positive |  |
| *Arthrobacter* |  | Positive |  |
| *Brachybacterium* |  | Positive |  |
| *Brevibacterium* |  | Positive |  |
| *Aeromicrobium* |  | Positive |  |
| *Propionibacterium* |  | Positive |  |
| *Nocardia* |  | Positive |  |
| *Corynebacterium* |  | Positive |  |
| *Chryseobacterium* | Bacteroidetes | Negative |  |
| *Elizabethkingia* |  | Negative |  |
| *Prevotella* |  | Negative |  |
| *Pedobacter* |  | Negative |  |
| *Sediminibacterium* |  | Negative |  |
| *Arcobacter* | Proteobacteria | Negative |  |
| *Aquabacterium* |  | Negative |  |
| *Achromobacter* |  | Negative |  |
| *Advenella* |  | Negative |  |
| *Cupriavidus* |  | Negative |  |
| *Ralstonia* |  | Negative |  |
| *Polynucleobacter* |  | Negative |  |
| *Burkholderia* |  | Negative |  |
| *Herbaspirillum* |  | Negative |  |
| *Comamonas* |  | Negative |  |
| *Pelomonas* |  | Negative |  |
| *Methylophilus* |  | Negative |  |
| *Uruburuella* |  | Negative |  |
| *Methylobacterium* |  | Negative |  |
| *Brevundimonas* |  | Negative |  |
| *Sphingomonas* |  | Negative |  |
| *Paracoccus* |  | Negative |  |
| *Sulfitobacter* |  | Negative |  |
| *Acinetobacter* |  | Negative |  |
| *Enhydrobacter* |  | Negative |  |
| *Pseudomonas* |  | Negative |  |
| *Ectothiorhodospira* |  | Negative |  |
| *Thiofaba* |  | Negative |  |
| *Halomonas* |  | Negative |  |
| *Providencia* |  | Negative |  |
| *Serratia* |  | Negative |  |
| *Proteus* |  | Negative |  |
| *Luteimonas* |  | Negative |  |
| *Thermomonas* |  | Negative |  |
| *Stenotrophomonas* |  | Negative |  |
| *Salinivibrio* |  | Negative |  |
| *Vibrio* |  | Negative |  |
| *Photobacterium* |  | Negative |  |
| *Aeromonas* |  | Negative |  |
| *Aliidiomarina* |  | Negative |  |
| *Thiothrix* |  | Negative |  |
| *Fervidobacterium* | Thermotogae | Negative |  |
| *Sulfurihydrogenibium* | Aquificae | Negative |  |
| *Peptoniphilus* | Firmicutes | Positive |  |
| *Dethiosulfatibacter* |  | Negative |  |
| *Streptococcus* |  | Positive |  |
| *Enterococcus* |  | Positive |  |
| *Aerococcus* |  | Positive |  |
| *Alkalibacterium* |  | Positive |  |
| *Geobacillus* |  | Positive |  |
| *Anoxybacillus* |  | Positive |  |
| *Brevibacillus* |  | Positive |  |
| *Salinicoccus* |  | Positive |  |
| *Staphylococcus* |  | Positive |  |
| *Deinococcus* | Deinococcus-Thermus | Positive |  |
| *Thermus* |  | Negative |  |
| *Chloroflexus* | Chloroflexi | Negative |  |

**Table S12D. Genera identified after incubating the Shivlinga inoculum in MMST medium at 55 ^O^C. The cultured metagenome was prepared after 16 hours of incubation when OD_600_ of the spent medium was 0.4 and pH 5.5. V3 regions of all bacterial 16S rRNA genes were PCR-amplified and sequenced on an Ion PGM.**

| **Genus** | **Phylum** | **Gram Stain of the type strain** | **Reference** |
| --- | --- | --- | --- |
| *Hydrogenophaga* | Proteobacteria | Negative |  |
| *Methylobacterium* |  | Negative |  |
| *Anoxybacillus* | Firmicutes | Positive |  |
| *Paenibacillus* |  | Positive |  |

| **Genus** | **Phylum** | **Gram Stain of the type strain** | **Reference** |
| --- | --- | --- | --- |
| *Microbacterium* | Actinobacteria | Positive |  |
| *Halomonas* | Proteobacteria | Negative |  |
| *Methylophaga* |  | Negative |  |
| *Anoxybacillus* | Firmicutes | Positive |  |
| *Bacillus* |  | Positive |  |
| *Terribacillus* |  | Positive |  |
| *Meiothermus* | Deinococcus-Thermus | Negative |  |

**Table S13A.** Genera identified after incubating the Paniphala Fountain inoculum in R2A medium at 30 ^O^C. The cultured metagenome was prepared after 12 hours of incubation when OD_600_ of the spent medium was 0.8. V3 regions of all bacterial 16S rRNA genes were PCR-amplified and sequenced on an Ion PGM.

**Table S13B. Genera identified after incubating the Paniphala Fountain inoculum in R2A medium at 55 ^O^C. The cultured metagenome was prepared after 12 hours of incubation when OD_600_ of the spent medium was 0.8. V3 regions of all bacterial 16S rRNA genes were PCR-amplified and sequenced on an Ion PGM.**

| **Genus** | **Phylum** | **Gram Stain of the type strain** | **Reference** |
| --- | --- | --- | --- |
| *Tepidimonas* | Proteobacteria | Negative |  |
| *Rhodobacter* |  | Negative |  |
| *Halomonas* |  | Negative |  |
| *Silanimonas* |  | Negative |  |
| *Methylophaga* |  | Negative |  |
| *Anoxybacillus* | Firmicutes | Positive |  |

**Table S13C. Genera identified after incubating the Paniphala Fountain inoculum in MMST medium at 30 ^O^C. The cultured metagenome was prepared after 72 hours of incubation when OD_600_ of the spent medium was 0.4 and pH 5.5. V3 regions of all bacterial 16S rRNA genes were PCR-amplified and sequenced on an Ion PGM.**

| **Genus** | **Phylum** | **Gram Stain of the type strain** | **Reference** |
| --- | --- | --- | --- |
| *Proteiniphilum* | Bacteroidetes | Negative |  |
| *Bacillus* | Firmicutes | Positive |  |
| *Paenibacillus* |  | Positive |  |

**Table S13D. Genera identified after incubating the Paniphala Fountain inoculum in MMST medium at 55 ^O^C. The cultured metagenome was prepared after 48 hours of incubation when OD_600_ of the spent medium was 0.4 and pH 5.5. V3 regions of all bacterial 16S rRNA genes were PCR-amplified and sequenced on an Ion PGM.**

| **Genus** | **Phylum** | **Gram Stain of the type strain** | **Reference** |
| --- | --- | --- | --- |
| Thermomonas | Proteobacteria | Negative |  |
| Anoxybacillus | Firmicutes | Positive |  |

**Table S14A.** Genera identified after incubating the Agnikunda inoculum in R2A medium at 30 ^O^C. The cultured metagenome was prepared after 16 hours of incubation when OD_600_ of the spent medium was 0.8. V3 regions of all bacterial 16S rRNA genes were PCR-amplified and sequenced on an Ion PGM.

| **Identifiedgenera** | **Phylum to which the**  **genera were affiliated** | **Gram stain of the type strain** | **Reference for Gram stain type** |
| --- | --- | --- | --- |
| *Bacillus* | Firmicutes | Positive |  |

**Table S14B.** Genera identified after incubating the Agnikunda inoculum in R2A medium at 55^O^C. The cultured metagenome was prepared after 16 hours of incubation when OD_600_ of the spent medium was 0.8. V3 regions of all

bacterial 16S rRNA genes were PCR-amplified and sequenced on an Ion PGM.

| **Identifiedgenera** | **Phylum to which the**  **genera were affiliated** | **Gram stain of the type strain** | **Reference for growth temperature(s) and Gram stain type** |
| --- | --- | --- | --- |
| *Bacillus* | Firmicutes | Positive |  |
| *Thermoactinomyces* |  | Positive |  |

| **Identified genera** | **Phylum to which the**  **genera were affiliated** | **Gram stain of the type strain** | **Reference for Gram stain type** |
| --- | --- | --- | --- |
| *Bacillus* | Firmicutes | Positive |  |
| *Planococcus* |  | Positive |  |
| *Thermus* | Deinococcus-Thermus | Negative |  |
| *Thiofaba* | Proteobacteria | Negative |  |
| *Sulfurihydrogenibium* | Aquificae | Negative |  |

**Table S14C.** Genera identified after incubating the Agnikunda inoculum in MMST medium at 30^O^C. The cultured metagenome was prepared after 48 hours of incubation when OD_600_ of the spent medium was 0.4 and pH 5.0. V3 regions of all bacterial 16S rRNA genes were PCR-amplified and sequenced on an Ion PGM.

**Table S14D.** Genera identified after incubating the Agnikunda inoculum in MMST medium at 55^O^C. The cultured metagenome was prepared after 96 hours of incubation when OD_600_ of the spent medium was 0.3 and pH 6.5. V3 regions of all bacterial 16S rRNA genes were PCR-amplified and sequenced on an Ion PGM.

| **Identified genera** | **Phylum to which the**  **genera were affiliated** | **Gram stain of the type strain** | **Reference for Gram stain type** |
| --- | --- | --- | --- |
| *Brachybacterium* | Actinobacteria | Positive |  |
| *Leifsonia* |  | Positive |  |
| *Nocardioides* |  | Positive |  |
| *Propionibacterium* |  | Positive |  |
| *Sediminibacterium* | Bacteroidetes | Negative |  |
| *Advenella* | Proteobacteria | Negative |  |
| *Ralstonia* |  | Negative |  |
| *Burkholderia* |  | Negative |  |
| *Hydrogenophaga* |  | Negative |  |
| *Methylophilus* |  | Negative |  |
| *Hyphomicrobium* |  | Negative |  |
| *Pedomicrobium* |  | Negative |  |
| *Methylobacterium* |  | Negative |  |
| *Brevundimonas* |  | Negative |  |
| *Sphingomonas* |  | Negative |  |
| *Paracoccus* |  | Negative |  |
| *Acinetobacter* |  | Negative |  |
| *Enhydrobacter* |  | Negative |  |
| *Pseudomonas* |  | Negative |  |
| *Thermomonas* |  | Negative |  |
| *Thermotoga* | Thermotogae | Negative |  |
| *Fervidobacterium* |  | Negative |  |
| *Caldicellulosiruptor* | Firmicutes | Negative |  |
| *Anoxybacillus* |  | Positive |  |
| *Bacillus* |  | Positive |  |
| *Aeribacillus* |  | Positive |  |
| *Brevibacillus* |  | Positive |  |
| *Thermus* | Deinococcus-Thermus | Negative |  |
| *Thermodesulfovibrio* | Nitrospirae | Negative |  |
| *Dictyoglomus* | Dictyoglomi | Negative |  |

| **Enrichment in R2A medium** | | | | **Enrichment in MMST medium** | | | |
| --- | --- | --- | --- | --- | --- | --- | --- |
| **Incubation**  **at 30 ^O^C** | | **Incubation**  **at 55 ^O^C** | | **Incubation**  **at 30 ^O^C** | | **Incubation at 55 ^O^C** | |
| **+V** | **-V** | **+V** | **-V** | **+V** | **-V** | **+V** | **-V** |
| **0.8 OD_600_ / 16 h**  Total no. of OTUs detected: 226  (SRR1954990)  Ubact, 16;  *Prote*, 200;  *Bact, 10*  Genus-level diversity data is given in **Supplementary Table S15A** | **0.8 OD_600_ / 16 h**  Total no. of OTUs detected: 310  (SRR1954988)  Ubact, 8; *Prote*, 233; *Firm*, *40*; *Bact, 22; Actn, 3; Cyan, 2; Aq, 1;*  *D-T*,1  Genus-level diversity data is given in **Supplementary Table S15B** | **0 OD_600_ / 480 h** | **0 OD_600_ / 480 h** | **5.2 pH; 0.4 OD_600_ / 48 h**  Total no. of OTUs detected: 268  (SRR1954993)  Ubact, 20;  *Prote*, 221;  *Bact,12; Cyan, 10; Fus, 3; Acd, 1; D-T, 1*  Genus-level diversity data is given in **Supplementary Table S15C** | **5.3 pH; 0.3 OD_600_ / 48 h**  Total no. of OTUs detected: 370  (SRR1954992)  Ubact, 22;  *Prote*, 296;  *Bact, 17; Firm*, *17; Actn,* 12; *Cyan*, 3; *Acd*, 2; *D*-*T*, 1  Genus-level diversity data is given in **Supplementary Table S15D** | **0 OD_600_ / 480 h** | **0 OD_600_ / 480 h** |

**Table S15.** V3 sequence-based bacterial taxonomic diversity detected upon incubating a lake water inoculum in R2A or MMST medium. End points of growths from where cultured metagenomes were prepared for PCR amplification and sequencing are described by the OD_600_ and pH of the spent media followed by the incubation time. V3 sequence files were deposited to the NCBI Sequence Read Archive (see SRA accession numbers in parenthesis) under the BioProject accession number PRJNA280244. Phylum-level classifications of the relevant OTU sets are shown here, while corresponding genus-level classifications are given in Supplementary Tables S15A through D. +V, growth medium supplemented with vancomycin (50 µg ml^-1^), -V, no vancomycin in growth medium.

U*Bact,* Unclassified *Bacteria*; *Prote,* Proteobacteria; *Bact,* Bacteroidetes; *Firm,* Firmicutes; *Actn,* Actinobacteria; *Cyan,* Cyanobacteria; *D-T,* Deinococcus-Thermus; *Acd,* Acidobacteria; *Aq,* Aquificae; Fus, Fusobacteria.

**Table S15A. Genera identified after incubating the EICB_LW_1 inoculum in R2A medium containing Vancomycin (50 µg ml^-1^) at 30 ^O^C. The cultured metagenome was prepared after 16 hours of incubation when OD_600_ of the spent medium was 0.8. V3 regions of all bacterial 16S rRNA genes were PCR-amplified, and sequenced on an Ion PGM.**

| **Identified genera** | **Phylum to which the**  **genera were affiliated** | **Gram stain of the type strain** | **Reference for Gram stain type** |
| --- | --- | --- | --- |
| *Chryseobacterium* | Bacteroidetes | Negative |  |
| *Emticicia* |  | Negative |  |
| *Flectobacillus* |  | Negative |  |
| *Sphingobacterium* |  | Negative |  |
| *Achromobacter* | Proteobacteria | Negative |  |
| *Cupriavidus* |  | Negative |  |
| *Burkholderia* |  | Negative |  |
| *Duganella* |  | Negative |  |
| *Herbaspirillum* |  | Negative |  |
| *Comamonas* |  | Negative |  |
| *Delftia* |  | Negative |  |
| *Acidovorax* |  | Negative |  |
| *Aquitalea* |  | Negative |  |
| *Brevundimonas* |  | Negative |  |
| *Acinetobacter* |  | Negative |  |
| *Pseudomonas* |  | Negative |  |
| *Halomonas* |  | Negative |  |
| *Dickeya* |  | Negative |  |
| *Klebsiella* |  | Negative |  |
| *Thermomonas* |  | Negative |  |
| *Aeromonas* |  | Negative |  |

**Table S15B. Genera identified after incubating the EICB_LW_1 inoculum in R2A medium at 30 ^O^C. The cultured metagenome was prepared after 16 hours of incubation when OD_600_ of the spent medium was 0.8. V3 regions of all bacterial 16S rRNA genes were PCR-amplified and sequenced on an Ion PGM.**

| **Identified genera** | **Phylum to which the**  **genera were affiliated** | **Gram stain of the type strain** | **Reference for Gram stain type** |
| --- | --- | --- | --- |
| *Agromyces* | Actinobacteria | Positive |  |
| *Cloacibacterium* | Bacteroidetes | Negative |  |
| *Chryseobacterium* |  | Negative |  |
| *Flectobacillus* |  | Negative |  |
| *Niabella* |  | Negative |  |
| *Burkholderia* | Proteobacteria | Negative |  |
| *Duganella* |  | Negative |  |
| *Comamonas* |  | Negative |  |
| *Delftia* |  | Negative |  |
| *Acidovorax* |  | Negative |  |
| *Aquitalea* |  | Negative |  |
| *Azospira* |  | Negative |  |
| *Pleomorphomonas* |  | Negative |  |
| *Azospirillum* |  | Negative |  |
| *Novispirillum* |  | Negative |  |
| *Rhodobacter* |  | Negative |  |
| *Acinetobacter* |  | Negative |  |
| *Pseudomonas* |  | Negative |  |
| *Dickeya* |  | Negative |  |
| *Serratia* |  | Negative |  |
| *Escherichia* |  | Negative |  |
| *Klebsiella* |  | Negative |  |
| *Thermithiobacillus* |  | Negative |  |
| *Thermomonas* |  | Negative |  |
| *Aeromonas* |  | Negative |  |
| *Tolumonas* |  | Negative |  |
| *Sulfurihydrogenibium* | Aquificae | Negative |  |
| *Streptococcus* | Firmicutes | Positive |  |
| *Lactococcus* |  | Positive |  |
| *Enterococcus* |  | Positive |  |
| *Weissella* |  | Positive |  |
| *Geobacillus* |  | Positive |  |
| *Anoxybacillus* |  | Positive |  |
| *Bacillus* |  | Positive |  |
| *Paenibacillus* |  | Positive |  |
| *Deinococcus* | Deinococcus-Thermus | Negative |  |

**able S15C. Genera identified after incubating the EICB_LW_1 inoculum in MMST medium containing Vancomycin (50 µg ml^-1^) at 30 ^O^C. The cultured metagenome was prepared after 48 hours of incubation when OD_600_ of the spent medium was 0.4 and pH 5.2. V3 regions of all bacterial 16S rRNA genes were PCR-amplified and sequenced on an Ion PGM.**

| **Genera** | **Phylum** | **Gram Stain of the type strain** | **Reference** |
| --- | --- | --- | --- |
| *Chryseobacterium* | Bacteroidetes | Negative |  |
| *Runella* |  | Negative |  |
| *Arcobacter* | Proteobacteria | Negative |  |
| *Achromobacter* |  | Negative |  |
| *Alcaligenes* |  | Negative |  |
| *Polynucleobacter* |  | Negative |  |
| *Burkholderia* |  | Negative |  |
| *Herbaspirillum* |  | Negative |  |
| *Comamonas* |  | Negative |  |
| *Delftia* |  | Negative |  |
| *Curvibacter* |  | Negative |  |
| *Acidovorax* |  | Negative |  |
| *Hydrogenophaga* |  | Negative |  |
| *Methylobacillus* |  | Negative |  |
| *Methylovorus* |  | Negative |  |
| *Methylophilus* |  | Negative |  |
| *Azonexus* |  | Negative |  |
| *Bosea* |  | Negative |  |
| *Rhodoblastus* |  | Negative |  |
| *Rhizobium* |  | Negative |  |
| *Methylocystis* |  | Negative |  |
| *Ancylobacter* |  | Negative |  |
| *Microvirga* |  | Negative |  |
| *Phenylobacterium* |  | Negative |  |
| *Azospirillum* |  | Negative |  |
| *Dongia* |  | Negative |  |
| *Roseomonas* |  | Negative |  |
| *Acinetobacter* |  | Negative |  |
| *Pseudomonas* |  | Negative |  |
| *Rheinheimera* |  | Negative |  |
| *Thiovirga* |  | Negative |  |
| *Providencia* |  | Negative |  |
| *Luteibacter* |  | Negative |  |
| *Stenotrophomonas* |  | Negative |  |
| *Aeromonas* |  | Negative |  |
| *Tolumonas* |  | Negative |  |
| *Meiothermus* | Deinococcus-Thermus | Negative |  |

**Table S15D. Genera identified after incubating the EICB_LW_1 inoculum in MMST medium at 30 ^O^C. The cultured metagenome was prepared after 48 hours of incubation when OD_600_ of the spent medium was 0.3 and pH 5.3. V3 regions of all bacterial 16S rRNA genes were PCR-amplified and sequenced on an Ion PGM.**

| **Genera** | **Phylum** | **Gram Stain of the type strain** | **Reference** |
| --- | --- | --- | --- |
| *Arthrobacter* | Actinobacteria | Positive |  |
| *Demequina* |  | Positive |  |
| *Flavobacterium* | Bacteroidetes | Negative |  |
| *Cloacibacterium* |  | Negative |  |
| *Bacteroides* |  | Negative |  |
| *Parabacteroides* |  | Negative |  |
| *Sphingobacterium* |  | Negative |  |
| *Arcobacter* | Proteobacteria | Negative |  |
| *Pandoraea* |  | Negative |  |
| *Polynucleobacter* |  | Negative |  |
| *Burkholderia* |  | Negative |  |
| *Comamonas* |  | Negative |  |
| *Delftia* |  | Negative |  |
| *Curvibacter* |  | Negative |  |
| *Acidovorax* |  | Negative |  |
| *Malikia* |  | Negative |  |
| *Hydrogenophaga* |  | Negative |  |
| *Limnohabitans* |  | Negative |  |
| *Methylobacillus* |  | Negative |  |
| *Methylovorus* |  | Negative |  |
| *Methylophilus* |  | Negative |  |
| *Vogesella* |  | Negative |  |
| *Thauera* |  | Negative |  |
| *Azoarcus* |  | Negative |  |
| *Azonexus* |  | Negative |  |
| *Bosea* |  | Negative |  |
| *Methylocystis* |  | Negative |  |
| *Pleomorphomonas* |  | Negative |  |
| *Phyllobacterium* |  | Negative |  |
| *Ancylobacter* |  | Negative |  |
| *Hyphomonas* |  | Negative |  |
| *Brevundimonas* |  | Negative |  |
| *Azospirillum* |  | Negative |  |
| *Roseomonas* |  | Negative |  |
| *Rhodobacter* |  | Negative |  |
| *Rhodobaca* |  | Negative |  |
| *Acinetobacter* |  | Negative |  |
| *Pseudomonas* |  | Negative |  |
| *Rheinheimera* |  | Negative |  |
| *Thiovirga* |  | Negative |  |
| *Thermomonas* |  | Negative |  |
| *Stenotrophomonas* |  | Negative |  |
| *Pseudoxanthomonas* |  | Negative |  |
| *Zobellella* |  | Negative |  |
| *Aeromonas* |  | Negative |  |
| *Alishewanella* |  | Negative |  |
| *Shewanella* |  | Negative |  |
| *Enterococcus* | Firmicutes | Positive |  |
| *Brevibacillus* |  | Positive |  |
| *Exiguobacterium* |  | Positive |  |
| *Chryseomicrobium* |  | Positive |  |
| *Lysinibacillus* |  | Positive |  |
| *Meiothermus* | Deinococcus-Thermus | Negative |  |

**References cited in Supplementary Tables**
